# Rapid sympatric ecological differentiation of crater lake cichlid fishes within historic times

**DOI:** 10.1186/1741-7007-8-60

**Published:** 2010-05-12

**Authors:** Kathryn R Elmer, Topi K Lehtonen, Andreas F Kautt, Chris Harrod, Axel Meyer

**Affiliations:** 1 Lehrstuhl für Zoologie und Evolutionsbiologie, Department of Biology, University of Konstanz, Universitätstrasse 10, 78457 Konstanz, Germany; 2 School of Biological Sciences, Monash University, Victoria 3800, Australia; 3 Department of Evolutionary Genetics, Max Planck Institute for Limnology, Postfach 165, 24302 Plön, Germany; 4 Current Address: School of Biological Sciences, Queen's University Belfast, Medical Biology Centre, 97 Lisburn Road, Belfast BT9 7BL, UK

## Abstract

**Background:**

After a volcano erupts, a lake may form in the cooled crater and become an isolated aquatic ecosystem. This makes fishes in crater lakes informative for understanding sympatric evolution and ecological diversification in barren environments. From a geological and limnological perspective, such research offers insight about the process of crater lake ecosystem establishment and speciation. In the present study we use genetic and coalescence approaches to infer the colonization history of Midas cichlid fishes (*Amphilophus *cf. *citrinellus*) that inhabit a very young crater lake in Nicaragua-the *ca*. 1800 year-old Lake Apoyeque. This lake holds two sympatric, endemic morphs of Midas cichlid: one with large, hypertrophied lips (~20% of the total population) and another with thin lips. Here we test the associated ecological, morphological and genetic diversification of these two morphs and their potential to represent incipient speciation.

**Results:**

Gene coalescence analyses [11 microsatellite loci and mitochondrial DNA (mtDNA) sequences] suggest that crater lake Apoyeque was colonized in a single event from the large neighbouring great lake Managua only about 100 years ago. This founding in historic times is also reflected in the extremely low nuclear and mitochondrial genetic diversity in Apoyeque. We found that sympatric adult thin- and thick-lipped fishes occupy distinct ecological trophic niches. Diet, body shape, head width, pharyngeal jaw size and shape and stable isotope values all differ significantly between the two lip-morphs. The eco-morphological features pharyngeal jaw shape, body shape, stomach contents and stable isotopes (δ^15^N) all show a bimodal distribution of traits, which is compatible with the expectations of an initial stage of ecological speciation under disruptive selection. Genetic differentiation between the thin- and thick-lipped population is weak at mtDNA sequence (*F*_ST _= 0.018) and absent at nuclear microsatellite loci (*F*_ST _< 0.001).

**Conclusions:**

This study provides empirical evidence of eco-morphological differentiation occurring very quickly after the colonization of a new and vacant habitat. Exceptionally low levels of neutral genetic diversity and inference from coalescence indicates that the Midas cichlid population in Apoyeque is much younger (*ca*. 100 years or generations old) than the crater itself (*ca*. 1 800 years old). This suggests either that the crater remained empty for many hundreds of years after its formation or that remnant volcanic activity prevented the establishment of a stable fish population during the early life of the crater lake. Based on our findings of eco-morphological variation in the Apoyeque Midas cichlids, and known patterns of adaptation in Midas cichlids in general, we suggest that this population may be in a very early stage of speciation (incipient species), promoted by disruptive selection and ecological diversification.

## Background

Since Darwin and Wallace, the study of island inhabitants has greatly influenced evolutionary and ecological research [1]. In many respects, crater lakes are the aquatic equivalent to islands. Like islands, the small size, distinct boundaries, simplified biota, young age, geographical isolation and frequently well-known geological history of crater lakes makes them well-suited to study the diversification of sister taxa [1-3]. The physical isolation of crater lakes means that after the first seeding by a colonizing lineage evolution may proceed rapidly *in situ*. Indeed, crater lakes have provided the most compelling examples of sympatric ecological speciation of fishes, particularly cichlid fishes [4-7]. Intralacustrine speciation in depauperate habitats (for example, postglacial lakes and crater lakes) typically proceeds due to disruptive ecological selection [8], though the rapidity, strength and completeness of this process depends on a number of population and environmental factors [5,9-11]. Well-known examples of this phenomenon include the benthic and planktivorous populations of sticklebacks [12,13], introduced salmon populations [14] and whitefish [15,16] in isolated lakes.

Cichlid fishes are pre-eminent non-model organisms for the study of ecological speciation because of their trophic polymorphism and rapid evolution [17,18]. Mesoamerican fishes of the Midas cichlid species complex (the *Amphilophus citrinellus *species group) are particularly variable in trophic characteristics such as body shape and pharyngeal jaw morphology, as well as maintaining a striking colour polymorphism [19-21] (for a review of the crater lakes and species complex see [22]). One of the most renowned examples of ecological differentiation in the species complex is the elongate, open-water species *Amphilophus zaliosus*, which evolved from a high-bodied benthic ancestor by sympatric speciation within Nicaragua's oldest crater lake, Lake Apoyo [4,20]. A similarly elongate but evolutionarily independent endemic species is also found in crater lake Xiloá [22-25]. An additional trophic polymorphism of Midas cichlids is fleshy, hypertrophied lips, which is believed to be involved in harvesting invertebrates from between cracks and recesses [20]. This character is absent from most crater lake Midas cichlid populations [20,22,26] and is best known from *Amphilophus labiatus*, a species that occurs in the two largest and shallowest lakes in the region, the great lakes Managua and Nicaragua [20]. Apoyeque is the only crater lake that harbours a sizeable population of Midas cichlids with hypertrophied lips; the origin and diversity of this population is the focus of our current study.

**Figure 1:**
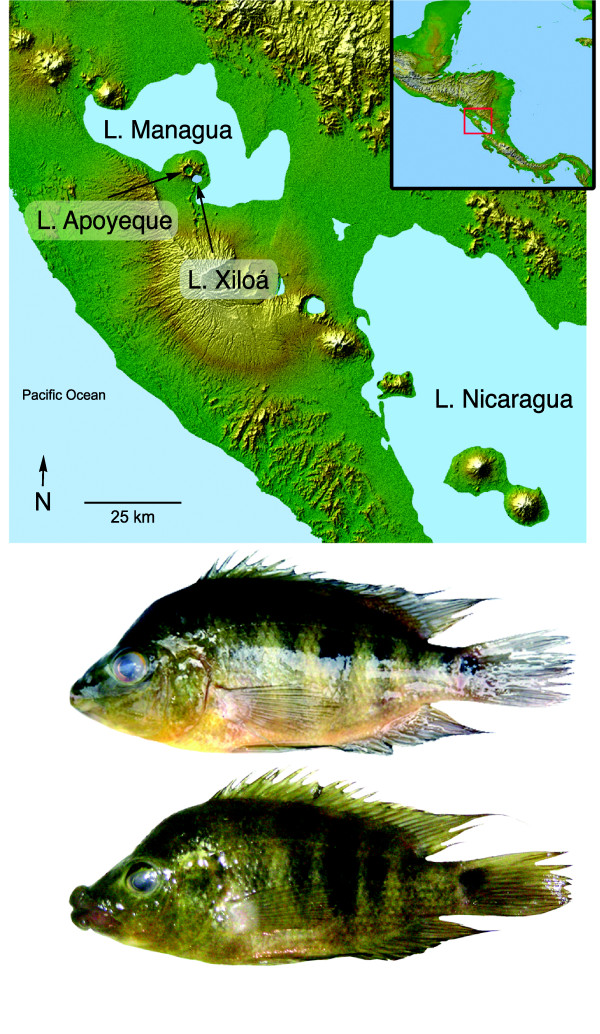
**Relief map of Nicaragua showing the relevant lakes**. Crater lake Apoyeque is located beside the great Lake Managua and the crater Lake Xiloá, in western Nicaragua, Central America. Two morphs of Midas cichlid are found in Apoyeque: one with fleshy lips (upper individual; 'thick-lipped') and the other with thin, normal *A. citrinellus *lips (lower individual; 'thin-lipped').

Western Nicaragua is underlain by an extremely active volcanism that has resulted in at least eight crater lakes that exist today (reviewed in [22]). One of the youngest of these is Apoyeque (Figure 1). The volcano last erupted only about 1800 years ago [27] and then filled with ground- and rainwater to become a crater lake. The lake is small (2.5 km^2^) yet deep (110 m) [28,29] and, because of its steep cone shape, has a fairly small littoral zone (0.9 km^2^) [30]. 'Apoyeque' means 'salty water' in the regional Náhuatl language and the lake is so-named because of its high mineral content [31]. Located on the Chiltepe Peninsula, Apoyeque lies next to crater lake Xiloá and great lake Managua and yet its high crater walls (400 m) and lack of water connection make it completely aquatically isolated.

Despite the crater lake Apoyeque's young age and isolation, it is home to an abundant population of Midas cichlids (*Amphilophus *cf. *citrinellus*). There are two phenotypes living in the lake: one with normal *citrinellus *thin lips ('thin-lipped') and one with pronounced fleshy, or hypertrophied, lips ('thick-lipped'; Figure 1). The lake is otherwise depauperate in species, only housing some small live-bearing fishes (Family Poeciliidae) and, less abundantly, the predatory cichlid *Parachromis managuense *[30].

In the present study, we test whether the two phenotypes (morphs) of Midas cichlid in Apoyeque originated by sympatric differentiation promoted by ecological divergence. Given the intralacustrine diversification of Midas cichlids in other crater lakes, we hypothesize that this trait of thin- or thick- lips may be a novel or alternative axis of ecological differentiation exploited by crater lake cichlids. First, we infer the colonization history of crater lake Apoyeque using population genetic and coalescent approaches, in the context of neighbouring lakes Managua and Xiloá. We specifically assess evidence for a single or multiple colonization events and see whether the age of the population corresponds to the geological age of the lake. Second, we compare ecological (diet, trophic level and pharyngeal jaw shape) and morphological (body shape, head and body size) variation between thin- and thick-lipped fishes to see whether this character is associated with distinct niches, sex or maturity, or morphological difference. Third, we assess the role of disruptive natural selection in driving incipient speciation by comparing the distribution of phenotypes and genetic differentiation in Apoyeque's Midas cichlid population, with the expectation that ecological diversification should result in a bimodal distribution of ecologically relevant traits.

## Results

### Colonization and population genetics of Apoyeque and neighbouring lakes

#### Genetic diversity and differentiation

**Figure 2:**
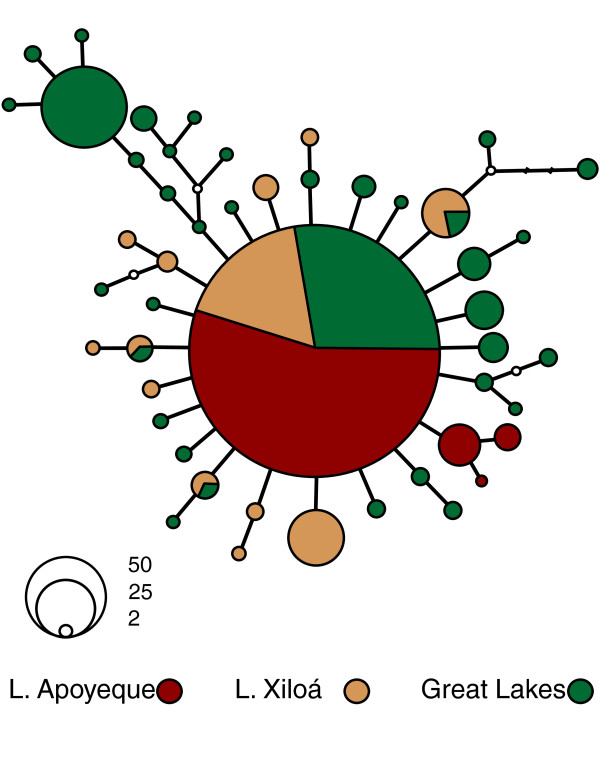
**A haplotype network of mtDNA sequences show that the most common haplotype in Midas cichlids is also the most common haplotype in Apoyeque**. Apoyeque has three haplotypes that are not shared with any other locality. Singleton haplotypes were included in the analysis but are not shown, except for one singleton from Apoyeque. Open circles represent missing or inferred haplotypes.

Using multiple approaches to assess genetic diversity with 11 nuclear microsatellite loci and mitochondrial DNA sequences, the Midas cichlid population in lake Apoyeque is the least genetically diverse relative to the Midas cichlid populations from nearby lakes (Table 1, Additional File 1, Additional File 2). Only four mitochondrial DNA (mtDNA) haplotypes were found among the 290 individuals sequenced from crater lake Apoyeque (Figure 2). The most abundant haplotype in Apoyeque is also found in Xiloá and in the great lakes Managua and Nicaragua (and was also the most common haplotype in previous Midas cichlid studies [4,24]). Three haplotypes unique to Apoyeque are much rarer (*n *= 14 individuals, all thin-lipped; *n *= 5 individuals, all thin-lipped; and *n *= 1 thick-lipped individual: see Genetic differentiation for further intralacustrine mtDNA analysis). Each of these Apoyeque-specific haplotypes is one-step apart from another Apoyeque haplotype and, to date, all are unique in the entire Midas cichlids species complex of Nicaragua (searched against 799 homologous Midas sequences, Genbank database accessed 27 April 2009), suggesting that they evolved *in situ *within the crater lake.

**Table 1:** Genetic diversity for Midas cichlid populations in three lakes: haplotype richness for mtDNA; observed heterozygosity, allelic richness, and private alleles for microsatellite markers (means ± standard deviations).


	**Crater Lake Apoyeque**	**Crater Lake Xiloá**	**Great Lake Managua**

Haplotype richness	0.131	0.574	0.851
Heterozygosity	0.632 ± 0.176	0.727 ± 0.214	0.785 ± 0.151
Allelic richness	5.97 ± 2.97	8.55 ± 3.71	11.24 ± 4.70
Private allelic richness	0.540 ± 0.532	1.14 ± 0.785	3.37 ± 1.83

The pronounced lack of microsatellite genetic variation within the Apoyeque Midas cichlid population, and its genetic distinctiveness from neighbouring lakes, can be visualized in the separation along axis 1 and the tight clustering of Apoyeque individuals in the Factorial Correspondence Analysis (Additional File 2). Midas cichlid fish populations in lakes Apoyeque, Xiloá and Managua are all significantly genetically differentiated from each other at mtDNA (*F*_ST _= 0.072 - 0.262) and microsatellite loci (*F*_ST _= 0.053 - 0.146; Additional File 3).

#### Coalescence inference of origin and time of colonization

Apoyeque could only have reasonably been colonized from either of its neighbouring lakes, Managua or Xiloá: all other lakes are geographically much more distant (Figure 1). Inferred from gene flow analyses in Migrate[32,33] (here a proxy for likely colonization), Lake Managua is the primary ancestral population of Apoyeque: migration from Managua to Apoyeque was estimated to be two to four times higher than the rate of migration from Xiloá to Apoyeque. Based on isolation-with-migration coalescence analyses (IMa), the divergence time between the populations of Apoyeque and its founding population from lake Managua (approximately equivalent to the time since colonization) was estimated to be about 100 years ago (89 years, 63 years and 132 years in three independent iterations; see Additional File 4 for likelihood values of all parameters). The effective population size of Apoyeque was estimated to be approximately 1000 times smaller than that of the ancestral population in great lake Managua (Additional File 4).

Apoyeque's extant Midas cichlid population probably originated from a single founding event. Historical migration (coalescence of genes from Managua to Apoyeque) was estimated to be negligible (~10 migrations/thousand generations) and there was a single probability peak for population divergence in the IMa analysis (Additional File 4).

### Eco-morphological variation within Apoyeque

#### Frequency of thin- and thick-lipped individuals

We collected exemplars of thin-and thick-lipped mature adults and juvenile fishes in Apoyeque. Twenty percent of the adult individuals were thick-lipped (42 of 214 sexed adults) and 24% of the juveniles were thick-lipped (19 of 80 specimens classified by dissection as juvenile). Additionally, 15 thick-lipped and 86 thin-lipped specimens were not classified to age or sex (see Methods and Additional File 5). There is no difference in the number of thick-lipped fishes in any of the four groupings (female, male, juvenile and not noted; *G*^2 ^= 5.15, df = 3, *P *= 0.161). Thus, from our total collection 19% of fishes were thick-lipped and 81% were thin-lipped.

#### Body shape and size

**Figure 3:**
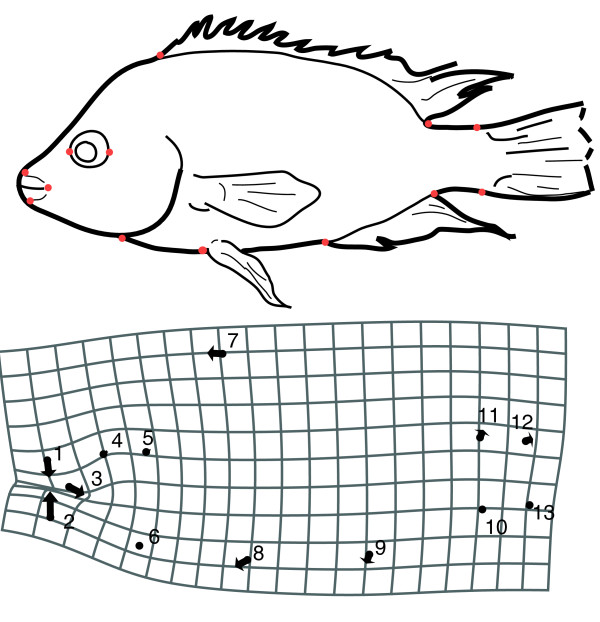
**Body shape variation between thin-lipped and thick-lipped Apoyeque Midas cichlids**. (a) The location of 13 landmarks to describe body shape. (b) Body shape differs significantly between thin- and thick-lipped fishes (*P *= 0.001). Here this is visualized (scale factor 4) as landmarks compared between the mean thick-lipped (circle; *n *= 36) and mean thin-lipped (arrow; *n *= 135) fishes. Body shapes remain different if the two landmarks describing lip thickness are excluded (*P *= 0.033) Body length does not differ between morphs (*P *= 0.34; see text for details).

Body shape of adult thin- and thick-lipped fishes differed significantly (Procrustes distance = 0.017, *P *= 0.001). Thick-lipped fish had narrower, longer mouths, were slightly deeper bodied and their body mass was shifted to the anterior relative to thin-lipped fishes (Figure 3). Shape was sufficiently different between the two lip morphs that most individuals could be correctly classified based on body shape alone (87% of thin-lipped fishes, 72% of thick-lipped fishes). Body shape remained significantly different when the lip landmarks (landmarks 1 and 2) were excluded (Procrustes distance = 0.013, *P *= 0.033).

The body size (standard length) of adult thin- and thick-lipped Midas cichlid fishes did not differ (two factor ANOVA, morph effect, *F*_1,202 _= 0.652, *P *= 0.42; Additional File 6).

Specifically focusing on head morphology, head width at the eyes was different for thin- and thick-lipped fishes (ANCOVA on log-transformed head width with log-transformed standard length as a covariate, morph effect, *F*_1,166 _= 7.09, *P *= 0.008; sex effect, *F*_1,166 _= 0.144, *P *= 0.71; morph × sex interaction, *F*_1,166 _= 0.857, *P *= 0.36). The two morphs had similar head widths at the snout (posterior point of the mouth: ANCOVA, morph effect, *F*_1,166 _= 0.051, *P *= 0.82; sex effect, *F*_1,166 _= 0.557, *P *= 0.46; morph × sex interaction, *F*_1,166 _= 1.62, *P *= 0.21; the nostrils: ANCOVA, morph effect, *F*_1,166 _= 0.006, *P *= 0.94; sex effect, *F*_1,166 _= 0.377, *P *= 0.54; morph × sex interaction, *F*_1,166 _= 0.275, *P *= 0.60). In other words, the snout width of thin- and thick-lipped fish does not differ but the head is thinner and more tapered in the thick-lipped fishes.

#### Lip size

Lip size did not differ between sexes (ANCOVA on log-transformed lip size with log-transformed standard length as a covariate, *F*_1,203 _= 0.158, *P *= 0.69). Thus, the extent of lip hypertrophy is not related to sex. However, adults (sexed males and females) had larger lips relative to their body size than juveniles (ANCOVA, *F*_1,280 _= 4.91, *P *= 0.028). Indeed, lip size clearly increased with body size (ANCOVA *F*_1,280 _= 179, *P *< 0.001). A plot of lip size by body length (including adult, juvenile and not noted specimens) demonstrates how the increase of lip size differs between the two morphs (Additional File 7; there is considerable overlap between thin- and thick-lipped fishes because we measured total lip area, not exclusively the protruding lip). The quadratic lines of best-fit differ markedly between thin- and thick-lipped fishes: the *y*-intercept is higher and the slope steeper in thick-lipped fishes. Thus lips get proportionally larger with maturity, age or size.

#### Diet

**Figure 4:**
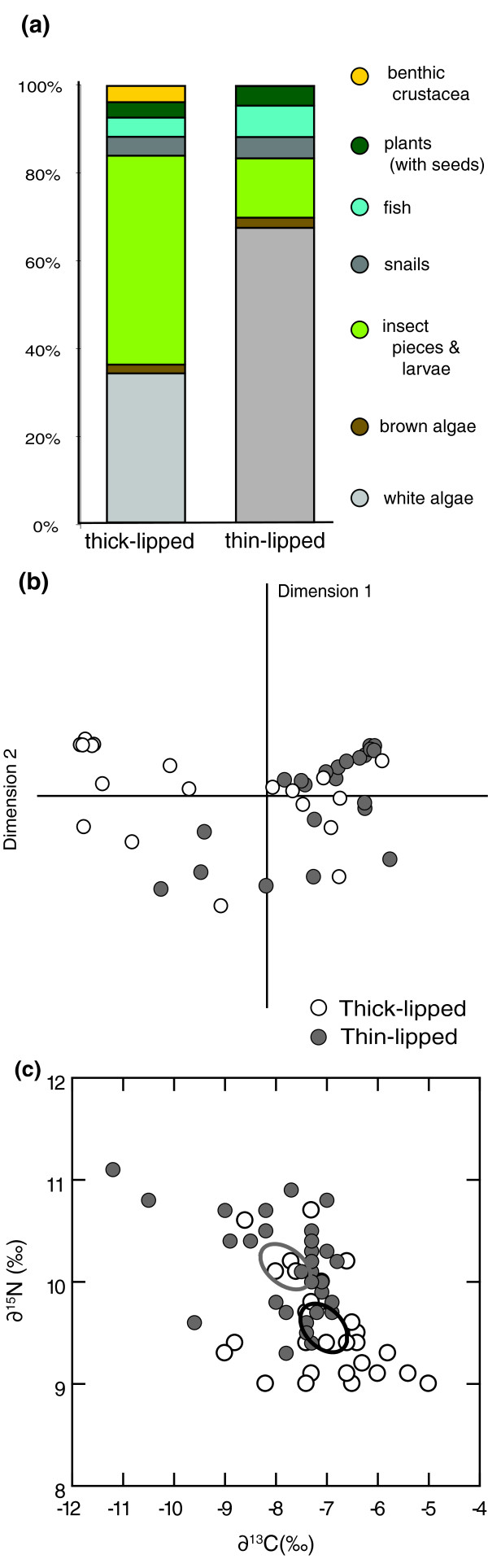
**Thick-lipped and thin-lipped Midas cichlids in Apoyeque differ quantitatively in diet and trophic niche**. (a) The proportion of gut contents per food category differs between thick- (*n *= 19) and thin-lipped (*n *= 22) fishes. (b) Multi-dimensional gut contents by morph displayed along the two primary axes. (c) Individual-level stable isotope values of δ^13^C and δ^15^N from thick-lipped (*n *= 29) and thin-lipped (*n *= 31) Apoyeque cichlids, with 95% confidence ellipses around the centroids.

The gut contents of adult thin- and thick-lipped fishes were markedly different, as implied by a significant interaction between the volume of prey type and morph (*G*^*2 *^= 35.5, *P *< 0.001), a Schoener's niche overlap index of 0.59 [34] and assortative clustering of the two morphs in multi-dimensional diet space (Figure 4a and 4b). The guts of thick-lipped fishes tended to contain more benthic crustacea, insect fragments and larvae while the guts of thin-lipped fishes contained more white algae (Oomycota), fish and snails (presumably the mollusc *Pyrgophorus coronatus *[35]).

Muscle tissue of adult thin- and thick-lipped fishes had different carbon (∂^13^C) and nitrogen (∂^15^N) signatures (non-parametric MANOVA: *F *= 13.0, *P *< 0.001) (Figure 4c). Thin-lipped fishes were typically slightly ^13^C depleted (median ± IQR = -7.4 ± 0.85 ‰) compared to thick-lipped fishes (-7.1 ± 0.95 ‰) and had higher ∂^15^N values (10.2 ± 0.7 ‰ versus 9.4 ± 0.7 ‰), suggesting that, on average, thin-lipped fishes fed at a slightly higher trophic level.

#### Lower pharyngeal jaws

**Figure 5:**
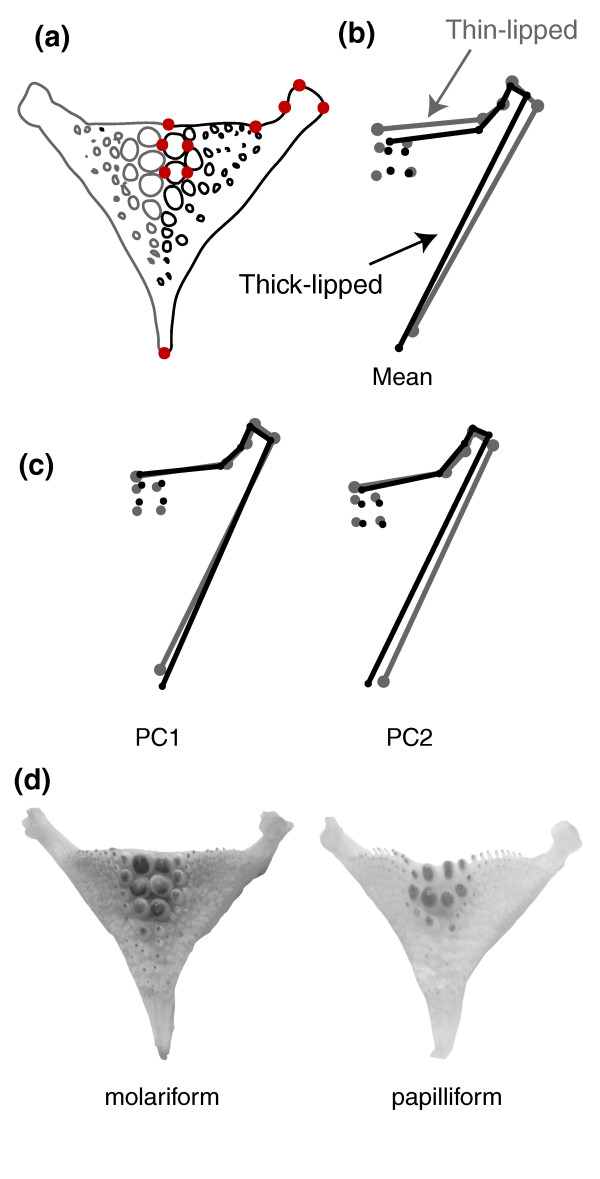
**Thick-lipped and thin-lipped Midas cichlids in Apoyeque differ in the shape of their pharyngeal jaws**. (a) Ten homologous landmarks describe jaw shape using one side. (b) Discriminant function analysis of mean shape of thick-lipped (black; *n *= 36) and thin-lipped (grey; *n *= 135) pharyngeal jaws (scale factor = 4). (c) The morphological variation associated with the first two principal component axes (scale factor = 4), responsible for most of the shape variation. (d) Exemplars of a molariform and papilliform pharyngeal jaws from Apoyeque Midas cichlids. Note the squatter, broader teeth and thicker horns in the more molariform jaw.

As the absolute pharyngeal jaw size significantly influenced its shape (thick-lipped *P *= 0.004, thin-lipped *P *< 0.001), we corrected for allometric effects using regression (see Methods). Pharyngeal jaw shape (including the two rear teeth; see Figure 5a for landmarks) differed significantly between thin- and thick-lipped fishes (Procrustes distance = 0.034, *P *< 0.001; Figure 5b). Thick-lipped fish have a narrower horn, longer jaw and two smaller rear teeth. Principal coordinates analysis corroborate these regions as having the most variation (Figure 5c). Thin-lipped fishes can be generally characterized as more molariform and thick-lipped fishes as more papilliform (an example of each pharyngeal jaw type is shown in Figure 5d).

### Disruptive selection and phenotypic differentiation

Quantitative eco-morphological traits are expected to have a bimodal distribution under disruptive selection. In order to test this, we first assessed whether the distribution of adult phenotypes for each eco-morphological trait fits a normal distribution (Additional File 8). Lip size (standardized to body length), body shape, lower pharyngeal jaw shape, stable isotope values of δ^13^C and gut contents significantly deviated from a single normal distribution (*P *< 0.001; Table 2). Stable isotope values of δ^15^N showed poor fit to a normal distribution (*P *< 0.06) and were included in further testing. For the traits of lip size, body shape, lower pharyngeal jaw shape and diet (gut contents), a bimodal distribution (equivalent to two normal distributions; see Methods) was strongly supported over a unimodal distribution by the corrected Akaike Information Criterion (AICc) values (Table 2, Additional File 8). Stable isotope values were less clearly bimodal: values of δ^ 15^N moderately supported better fit to a bimodal distribution while values of δ^13^C equally supported either a uni- or bimodal distribution.

**Table 2:** Statistical support for whether the distribution of phenotypes for eco-morphological traits is composed of one or two normal distributions.

Character	Data	Goodness-of-fit	AICc for unimodal	AICc for bimodal	ΔAICc	Supported distribution
Lip size	Standardized area	*P *< 0.0001	-139.83	-448.46	308.64	Bimodal (strong support)
Body shape	PC1	*P *< 0.0001	-486.00	-1037.57	551.57	Bimodal (strong support)
Pharyngeal jaw	PC1	*P *< 0.0002	-411.53	-868.68	457.15	Bimodal (strong support)
Diet	MDS 1	*P *< 0.0001	83.53	64.05	19.48	Bimodal (strong support)
Stable isotope (13C)	13C defatted	*P *< 0.0008	139.41	134.52	4.89	Unimodal = bimodal
Stable isotope (15N)	15N	*P *< 0.0556	71.65	64.50	7.16	Bimodal (moderate support)

Data are composed of raw or multivariate data. Low *P *values for goodness-of-fit suggest poor fit of the data to a single normal distribution. ΔAICc is the difference in model AICc for a single- compared to a two-component model. Levels of support for one model over the other are described in the Methods. Frequency histograms and normality plots can be found in Additional File 7.

AICc, Akaike Information Criterion; MDS, multidimensional scaling.

### Genetic differentiation within Apoyeque

There was no genetic differentiation at microsatellite loci between the population of thin- and thick-lipped fishes in Apoyeque (*F*_ST _< 0.001, *P *= 0.360). There was genetic differentiation in mtDNA haplotypes between the population of thin- and thick lipped fishes (*F*_ST _= 0.018, *P *= 0.064), though it is not strongly statistically significant.

## Discussion

### Rapid intralacustrine eco-morphological differentiation

Analyses of ecologically relevant traits indicate that thick- and thin-lipped fishes occupy two distinct niches in sympatry within the small and extremely young crater lake Apoyeque. The two morphs have a different diet, as reflected by Schoener's index (0.59) and a clustering into separate multi-dimensional diet space (Figure 4b). Thick-lipped fishes tend to have a diet richer in benthic crustacea and insect prey (Figure 4a). They are also enriched in δ^13^C and depleted in δ^15^N stable isotope values compared to thin-lipped fishes (Figure 4c), which may suggest a greater use of littoral prey [36]. The pharyngeal jaws of thick-lipped fishes are significantly thinner in shape, i.e. characteristically more papilliform (Figure 5). Papilliform-jawed Midas cichlids in other crater lakes are more insectivorous [4,37,38], as they also tend to be in Apoyeque, reflecting the established correlation between diet and jaw in cichlids. In contrast, thin-lipped fishes tend to be more molariform, with thicker jaws and broader teeth (Figure 5). Molariform jaw morphology is associated with more efficient snail crushing and a benthic diet in Midas cichlids [38,39] and cichlids generally [40], and also here in Apoyeque. We found that the shape of pharyngeal jaws was significantly affected by jaw size, which may support the hypothesis that pharyngeal jaws change shape over time depending on diet [39].

Lip hypertrophy is found in males and females at similar frequencies and the relative size of the lips does not differ between the sexes. Hence, lip hypertrophy is not a sex-linked character. Nor is lip hypertrophy a character that develops only after maturity: it was found at similar frequencies in adults and juveniles. However, lip size increases with body length, indicating that the hypertrophy may get more pronounced with age. The relationship of increasing lip size with body size is not the same for thin- and thick-lipped fishes. Instead, thick-lipped fishes show a much more dramatic increase of lip size with body size (Additional File 7). Therefore, it would be interesting to build upon our research by studying trophic variation at across life history stages, a possible role of plasticity or feedback between ecological niche and lip hypertrophy, and genetic influences on lip hypertrophy.

Although ecological differentiation between lip morphs is clear, overall body shape (Figure 3) and head widths differ between lip morphs only subtly, albeit significantly. Indeed, the body shape difference between other Midas cichlid species, such as syntopic thin-lipped *A. citrinellus *and thick-lipped *A. labiatus *in the great lakes Managua and Nicaragua, is much more pronounced [22,41] than what we found between the orthologous thin- and thick-lipped morphs in Apoyeque. In this regard, it is important to note that the thin- and thick-lipped Midas cichlids in Apoyeque are morphologically, genetically and geographically distinct from the Midas cichlids in lakes Managua and Nicaragua (this study and [22]), indicating that they are not simply allotopic populations of *A. citrinellus *and *A. labiatus*.

Disruptive selection in sympatry is expected to result in a bimodal distribution of quantitative eco-morphological traits. This is because intraspecific competition and negative frequency dependent selection result in higher fitness of extreme over intermediate phenotypes (reviewed in [42]). Such a pattern has been demonstrated under ecological speciation in nature but is notoriously difficult to quantitate (for example [43-46]). In Apoyeque Midas cichlids, lip size and a variety of eco-morphological traits that are reflective of trophic niche-body shape, pharyngeal jaw shape, stomach contents and δ^15^N-are all found at frequencies predicted under frequency-dependent fitness and disruptive selection. Specifically, these traits better fit a bimodal rather than unimodal distribution (Table 2, Additional File 8). Secondary contact could also result in a bimodal distribution of phenotype, but cichlid ecology, a single colonization event and no genetic evidence for secondary admixture (discussed below), suggests this is not the case in Apoyeque. Extensive empirical and theoretical research suggests that disruptive selection can drive multimodal eco-morphological differentiation and, with even partial non-random mating, result in incipient adaptive speciation [42,47,48].

Despite the bimodality of phenotype, we find no equivalent differentiation in nuclear genetic markers between thin- and thick-lipped Midas cichlids in Apoyeque (microsatellite *F*_ST _< 0.001, *P *= 0.360). This absence of differentiation may be due to very low population genetic polymorphism, a young population with younger divergence, or weak or absent assortative mating; field observations will be necessary. However, we do find population differentiation in the mtDNA haplotypes (*F*_ST _= 0.018, *P *= 0.064). While marginally statistically non-significant, this represents a substantial sympatric genetic differentiation within Apoyeque based on eco-morphology. To put it in context, this is one-third of the differentiation found among allopatric Midas cichlid populations of lakes Managua *vs*. Xiloá (*F*_ST _= 0.053, *P *< 0.001, Additional File 3). More striking is that the mtDNA divergence between morphs within Apoyeque exceeds that of the different thin- and thick-lipped species, *A. citrinellus *and *A. labiatus*, in the great lake Nicaragua (*F*_ST _= 0.004, *P *= non-significant [24] also based on mtDNA control region). It is presently unclear what processes are at work that would result in panmixia at nuclear loci and differentiation at mtDNA in this young, sympatric, genetically depauperate Midas cichlid population and this should be the focus of future research.

Our finding of eco-morphological differentiation with low genetic differentiation could suggest that lip morph is plasticity, polymorphism or incipient speciation. We discuss these alternatives below.

### Midas cichlid species and ecological traits

Hypertrophied lips are an overt species-specific character related to ecology in cichlids [49]. It has evolved independently in different environments and species complexes of various New World (for example [50]) and Old World cichlids ([51] and references therein; [17,52,53]). It is considered to be a diagnostic trait: among the scores of described cichlid species (1611 Cichlidae in FishBase.org, 5 November 2009), we know of none for which the character of hypertrophied lips is a described intraspecific polymorphism (though there is debate about *Crenicichla iguassuensis*, see [54,55]).

For example, the Nicaraguan great lake Midas cichlid species *A. citrinellus *and *A. labiatus *are discerned primarily by lip morphology, although they also differ in head shape, snout angle, feeding behaviour, anterior teeth, fin position and length [20,56], pharyngeal jaws [24,41] and body shape [22,41]-much like the two morphs of Midas cichlid in Apoyeque. In contrast to these differences, some meristic values overlap between *A. citrinellus *and *A. labiatus *and the extent of lip hypertrophy varies within populations [56]. Further, neutral genetic differentiation between those two species is low or absent [24]. This is a conundrum because young eco-morphological variants (for example, benthic or limnetic species) in various crater lakes can be discerned genetically (for example [4,25,57]). Thus, early researchers of Nicaragua's cichlids vacillated on the issue of polymorphism versus speciation but concluded that the weigh of evidence argued for two Midas cichlid species in the great lakes, *A. citrinellus *and *A. labiatus *[20,56] and all currently published research proceeds under the assumption that they are different species. In the laboratory, *A. labiatus *mate assortatively and resultant offspring have thick lips (KRE and AM unpublished data), suggesting that this trait is involved in sexual isolation by assortative mating and has a strong genetic component. Mate choice experiments between *A. citrinellus *and *A. labiatus *have, to our knowledge, never been published. Certainly, further research is needed on 'lippiness' and this is a research topic that we are currently undertaking from ecological, behavioural and genetic perspectives.

Therefore, although at present we cannot completely rule out that being thin- or thick-lipped is an intrapopulation polymorphism or plasticity in Apoyeque, we consider it quite unlikely. Instead, we suggest that the ecological differentiation between thick- and thin-lipped eco-morphs in Apoyeque represents a case of ecologically distinct, sympatric incipient sister species. Moreover, lips may be one of the very few examples for a 'magic trait' [58]-a trait that combines disruptive natural selection and assortative mating and that also (*sensu *[59]) might lead to speciation in sympatry. Low or absent genetic differentiation between morphs does not contradict incipient ecological speciation because it is an overly conservative estimator [60] and neither microsatellites nor mtDNA sequences robustly differentiate other species of Midas cichlid that differ in lip character [24]. It may be that the same ecological, genetic or behavioural factors responsible for low meristic and genetic divergence between *A. citrinellus *and *A. labiatus *in Nicaragua's great lakes are also at play in Apoyeque. Further research on the association of eco-morphological divergence, such as lip hypertrophy, with reproductive isolation is needed in all Midas cichlids.

In other Nicaraguan crater lakes, intralacustrine divergence of Midas cichlids has occurred with a different set of ecological traits-primarily body elongation [22]. In some cases this progressed to full species status with phenotypic, meristic and molecular divergence, such as in lakes Apoyo [4,57] and Xiloá [25,61]. Though thick-lipped Midas cichlids are rather common in Apoyeque (~20% of the population), they are found only sporadically in crater lake Masaya (personal observation, KRE, AM and [19]) and extremely rarely in crater lake Xiloá (personal observation, KRE, TKL and AM and [20,26]), and are believed to be completely absent in many crater lakes (for example, Apoyo, Asososca Managua and Asososca León). This suggests that hypertrophied lips is not the ancestral state in this species complex and instead may only arise under particular ecological conditions and genetic backgrounds.

### Crater lake colonization

The dearth of genetic variability sampled in Apoyeque Midas cichlids is consistent with coalescence analyses that indicated a very recent founding of Apoyeque (approximately 100 years or generations ago) by a single colonization event. The coalescent methods we employed here have been used to infer the age of African cichlid species because the recent speciation in many cichlid species hinders lineage-based phylogenetic methods [62,63]. Even if this method underestimates the colonization time, all geological and genetic evidence indicate that the Apoyeque population is extremely young (that is, maximally 1800 years old) and the most genetically depauperate of all the crater lake Midas cichlid populations [22].

How a high walled crater lake could be colonized by fishes is a tantalizing question for history. Some possibilities include being transported by birds (for example, ospreys, which fly moderate distances with fish in their talons), momentary subterranean aquatic connections caused by volcanic and tectonic movements or being flung in by hurricanes or funnel winds with water transfer between neighbouring lakes; spectacular though these phenomena seem, they have been reported scientifically for many years (reviewed in [64,65]). It is also possible that the population of fish in Apoyeque was seeded by humans, as is proposed for other lakes [66], but this seems less plausible because the lake is quite difficult to access and is not located next to any human settlement. The presence of a large population of small poeciliid fish in Apoyeque, which would not likely be purposely introduced for food or sport, argues that natural colonizations may be more likely than human introductions. Regardless of the historical mode of arrival, our data suggest that the extant population of Midas cichlid fishes colonized Apoyeque only once and very recently.

Is crater lake colonization rare? If crater lake Apoyeque is about 1800 years old, but the Midas cichlid population within is only about 100 years old, did the lake stand devoid of fish all the time in between? One possibility is that cycles of extinction and re-colonization may be relatively frequent in the early life of a crater lake. Occurrences such as remnant volcanic activity, fumerole activity [67] or periodic gas releases and rising of toxic waters [68,69] may exterminate a crater lake's fish fauna. For example, locals of the area report that Apoyeque occasionally 'boils' and 'all' of the fish die (personal communication to KRE). Such a fish kill was observed in January 2008 in Lake Masaya, though clearly not all fish died (personal observation, TKL). In that case, Apoyeque may have been colonized previously, followed by an extinction event, and the extant population only arrived within the past 100 years. Additional comparative phylogeographic investigations across lakes and species will help in addressing these questions, but ultimately a crater lake's colonization history may remain speculative.

### Founder events, novel habitats and speciation

Nicaraguan crater lake environments, being small, deep, clear, mineral-rich isolated waters, are very different from the large, shallow, turbid waters of the ancestral great lakes. This represents a major shift in ecological niche for Midas cichlids and may allow for the rapid evolution of new phenotypes [70,71]. To date, we have largely relied on models to suggest how long phenotypic divergence and speciation may take after colonisation of a new environment. Those estimates vary greatly: some model suggest 10 000 generations may be sufficient for the emergence of ecologically differentiated species under favourable conditions [5,72], or possibly much faster [60,73]. Based on field collected data, especially for fish, other authors [14-16,74-76] argue that ecological speciation or morphological diversification can occur on timescales of only tens to hundreds of generations. Here, we have demonstrated that ecological divergence can begin in sympatry on historic time scales of a minimum of 100 years.

## Conclusions

The results of our study suggest: (i) a case of ecological and morphological differentiation of sympatric Midas cichlids to exploit divergent niches, with associated weak population divergence at mtDNA; and (ii) that this eco-morphological divergence occurred very quickly after colonization of a crater lake habitat. Midas cichlids in Apoyeque are geographically and genetically isolated from neighbouring populations, and morphologically distinctive [22] and, thus, evolutionarily independent from other populations. Whether thin- and thick-lipped Midas cichlids in crater lake Apoyeque are a single polymorphic species or two incipient species cannot yet be determined with certainty but, based on cichlid biology in general and patterns of genetic and eco-morphological variation in Apoyeque in particular, we suggest that thin- and thick-lipped fishes may be sympatric incipient species.

## Methods

### Specimen collection

Specimens were collected in November and December 2007 using gill nets in lakes Apoyeque (12.15°N, 86.20°W) and Xiloá (12.13°N, 86.19°W) (Figure 1). Specimens from Managua were collected previously by AM and M Barluenga. Fish were digitally photographed in a standardized position. Maturity and sex were determined by dissection for a subset of specimens (see Additional File 5). Tissue and voucher specimens (whole fish or head) were collected for every specimen and stored in ethanol. Stomachs that held contents were collected and stored in ethanol. Apoyeque fishes were classified as having normal lips ('thin-lipped') or hypertrophied lips (if the upper lip protruded forward beyond the snout profile; 'thick-lipped') based on standardized photos (Figure 1). Although there is variation in the extent of hypertrophy of the 'thick-lipped' lips (within Apoyeque and across the Midas species complex [20,66]), the great majority of individuals were readily assignable.

A subset of adult specimens from Apoyeque was used to assess variation in pharyngeal jaw shape, body size, head width, and body shape (thick-lipped ♀ *n *= 23, thick-lipped ♂ *n *= 13, thin-lipped ♀ = 66, thin-lipped ♂ *n *= 69; Additional File 5). A more extensive group of specimens was used for lip size covariate analysis (pooled for the two morphs: ♀ *n *= 106, ♂ *n *= 100, juveniles *n *= 77) and the plot of lip size by body size (thin-lipped *n *= 309, thick-lipped *n *= 71; Additional File 5, Additional File 6, Additional File 7). The final microsatellite data set comprised: Apoyeque thin-lipped *n *= 317, Apoyeque thick-lipped *n *= 76, Xiloá *n *= 50, Managua *n *= 185 (Additional File 5). The mtDNA data set comprised: Apoyeque thin-lipped *n *= 232, Apoyeque thick-lipped *n *= 58, Xiloá *n *= 29, Managua *n *= 70 new or resequenced (Additional File 5). Juvenile and 'not noted' fishes were included in genetic analyses and the lip size plot but were not analysed for eco-morphological variation in order to avoid confounding effects of developmental stage on niche and body shape.

### Body size and shape variation

A single observer measured head width from voucher specimens using digital callipers at: (1) posterior point of mouth; (2) nostrils; and (3) posterior point of eye. Standard body length and the volume of the lower and upper lip (lip size) were measured in ImageJ v 1.41o [77]. In order to test the influence of sex or maturity on lip size, the effect of body size was standardized by including standard length as a covariate in the lip size analyses. Initially, we included the full model using log-transformed body size and lip size values. After the interaction term was found to be non-significant, it was removed and we proceeded testing for the main effects.

Thirteen landmarks to describe body shape were digitized from standardized photographs in TPSDig version 2.12 [78] (Figure 2) and analysed with classifier variables (sex, lip morph) in MorphoJ version 1.00j [79] using least-squares Procrustes superimposition [80] and geometric morphometric methods. Body shape analyses have been described elsewhere [22]. There was no significant influence of body size on body shape in any group (thick-lipped ♀ *P *= 0.76, thick-lipped ♂ *P *= 1.0, thin-lipped ♀ *P *= 0.62, thin-lipped ♂ *P *= 0.19; 10 000 permutations) so no allometric correction was applied. Body shapes of males and females do not differ (Procrustes distance = 0.008, *P *= 0.12) so sexes were subsequently combined.

### Ecological niche assessment

Lower pharyngeal jaws were extracted and cleaned in a solution of 10% trypsin for 17 h before being imaged using a desktop scanner. Jaws are an approximately symmetrical structure [81] so only one side was used to infer shape. Ten landmarks (approximately following [81]) were digitally assigned using TPSDig (Figure 5a). Jaw shape was analyzed in MorphoJ by the same approaches used for body shape. Male and female jaws do not differ in shape (Procrustes = 0.006, *P *= 0.79: allometry corrected) so sexes were subsequently combined.

Gut contents were categorized under a stereoscope (observer blind to fish's morph) for groupings benthic crustacea, plants and seeds, fish and fish parts, snail shells, insect pieces and larvae, brown algae, white algae [determined by polymerase chain reaction (PCR) to be Oomycota]. Volume of each category was determined by comparing it to a microlitre standard after 30 s centrifugation [82]. In order to test whether the two morphs differed in diet, we applied a generalized linear mixed model. Volume was used as a response variable with food category and morph as fixed effects. Identity code of each individual was entered as a random effect. The model was fitted using maximum likelihood and a likelihood ratio (*G*^*2*^) test was used to calculate the *P*-value for the interaction term between food category and morph. In order to improve the normality of the residual errors, the food volume values were log + 1 transformed prior to the analysis. In addition, we calculated Schoener's index of diet difference [83] and used monotonic multidimensional scaling (MDS) with Guttman loss function to scale pair-wise proportional similarities in gut content into a two-dimensional plot. Digested, unrecognizable material in the guts was excluded from all diet analyses.

Carbon (∂^13^C) and nitrogen (∂^15^N) stable isotope values were determined following [84]. Values of ∂^13^C were normalized for lipid content [85]. Non-parametric MANOVA in Past[86] was used to compare groups statistically.

Normality of each eco-morphological trait was assessed by plotting and observing a frequency histogram of values and normal probability plots (using raw data, PC1 or MDS1; Additional File 8) and the statistical test of goodness-of-fit to a normal distribution was calculated by Shapiro-Wilk W in JMP version 7 [87]. For all non-normally distributed traits, we tested whether the plots are best fit by a single normal distribution or a bimodal distribution using a two-component normal mixture model in discmixtureprogs version 0.5 mix2 http://www.bioss.ac.uk/~markb/mixtures/[88]. This method uses numerical integration to calculate mean, variance and proportion for each distribution by integration over a posterior density [88]. Bayesian mixture models were run for 500 iterations (bins) and convergence assessed by stabilized trace plots and a posterior distribution of theda and sigma values that started at, and returned to, zero or near-zero (not shown). The fit of a single-component model and a two-component model to our data was calculated with AICs (second-order, or corrected for sample size). The difference between models (ΔAICc) was calculated from the AICc for a single unimodal distribution minus the AICc for a two-component distribution (reviewed in [88,89]). We interpreted ΔAICc values following established guidelines: 0-5 are equivalent support for either model, 5-8 is moderate support for one model over the other, >8 is strong support for one model over the other. A negative ΔAICc value supports a single normal distribution and positive ΔAICc value supports a two-component distribution over a single-component [44,89].

### Mitochondrial DNA sequencing and analysis

Samples were sequenced for mtDNA using standard methods described elsewhere [22]. Diversities were calculated in Contrib version 1.02 [90] rarefied to the smallest sample size (Xiloá, *n *= 29). Sequences were collapsed into haplotypes ignoring missing data in DnaSP version 4 [91]. *F*_ST _estimates were calculated in Arlequin version 3.11 [92] using a Tamura [93] + gamma (unequal base frequencies, unequal substitution rates, alpha = 0.74) corrected distance matrix to approximate the best model of evolution that was inferred to be HKY [94] + I + G from Modeltest version 3.7 [95] in PAUP* version 4 [96]. Statistical power was determined by a 10 000 step Markov Chain method. Sequences from Apoyeque were searched in on-line nucleotide BLAST http://blast.ncbi.nlm.nih.gov/ for identical sequences (Identity = 100%) in the nr/nt database.

Using all newly determined sequences and all Midas cichlid complete control region sequences from nearby lakes Xiloá, Nicaragua, Managua, and Tisma Pond available in Genbank (*n *= 459; as of 19 Jan. 2009), a median-joining haplotype network [97] was calculated in Network version 4.5.1.0 http://www.fluxus-engineering.com. Transversions tend to be rarer in mitochondrial DNA and were, therefore, weighted three-times transitions. The MJ network was maximum parsimony post-processed to display the shortest tree compatible with the predictions of coalescence [98]. In order to simplify visualization, singleton haplotypes are not drawn in the network, except from Apoyeque.

### Microsatellite genotyping and analyses

Eleven microsatellite loci were amplified using standard PCR conditions: Abur151, Abur82 [99], M1M(=Acit1), M2(=Acit2), M7(=Acit3), M12(=Acit4) [100], Unh002 [101], Unh012, Unh013 [26], Burtkit F 474/R672 [102] and TmoM7 [103]. Fluorescently labelled fragments were analysed on an ABI3130XL (Applied Biosystems, CA, USA) and sized according to Rox 500 internal standard in GeneMapper version 4.0 (Applied Biosystems).

Genotyping quality for each population was assured by testing in Micro-Checker[104] with 5 000 randomizations and a 95% confidence interval. Any influence of null alleles was always low (Brookfield 1 [105] < 0.06), suspected in only two or fewer loci and with no consistent deviation across loci or populations, so all loci were retained. Expected heterozygosity, inbreeding coefficient *F*_IS _and inter-population *F*_ST _were calculated in Microsatellite Analyzer[106]. Rarefied allelic richness and private allelic richness (standardized to the lowest number of alleles) [107] were calculated in HP-Rare version 1.0 [108]. Hardy Weinberg exact tests and linkage disequilibrium were calculated in Genepop '007 [109,110]. Multiple tests were Bonferroni corrected. Genetic variation was analysed in a two-dimensional individual-based factorial correspondence analysis in Genetix[111].

### Coalescence analyses

The population (Managua or Xiloá) that founded Apoyeque was estimated from the microsatellite dataset using Migrate version 2.4.3 [32,33]. We applied the Bayesian inference [112] and restricted Apoyeque gene flow to immigration. Apoyeque was reduced to *n *= 200 to decrease disparity between population sample sizes, as such disparity can cause problems for parameter convergence. We applied a static heating scheme of four chains with the temperatures 10 000.00, 3.00, 1.50 and 1.00. Mutation rates varied between loci and were estimated from the data. Three independent runs were conducted, each consisting of 4 000 000 MCMC steps and a burn-in period of 100 000 steps. Albeit absolute posterior parameter estimates deviated slightly due to the stochastic nature of the coalescence analyses, all runs resulted in similar estimates. This suggests adequate convergence and a good match between the data and the models.

Divergence time between the founding population, Managua, and Apoyeque was calculated in IMa[113,114] using all mtDNA and microsatellite data combined. We used a geographic heating scheme with 60 concurrent chains and applied values of 0.9 and 0.95 for the heating parameters g1 and g2 to achieve adequate swapping among chains (> 0.4 even for low numbered chains). Each run began with a different random number seed and ran for 1 000 000 steps following a burn-in period of 250 000 steps. Mutation rate is estimated at 5 × 10^-4 ^per locus per year [115]. Migration rates and population sizes were restricted based on priors that generated generally well-behaved posterior distributions in optimizing runs (q1 1, q2 10, qA 200, m1 100, m2 5). We initially restricted divergence time to 2 000 year (*t *1) to match Apoyeque's geological age but this resulted in an unstable peak at the maximal divergence, probably due to ancestral polymorphism. A peak at about 100 years was resilient to varying maximal divergence values. Three runs with maximal divergence 10 000 years (*t *5) were iterated with different starting seeds to confirm consistency of the results and they all converged on similar values with the highest posterior density between 60 and 150 years.

## Abbreviations

AICc: Akaike Information Criterion; IMA: isolation-with-migration coalescence analysis; MDS: multidimensional scaling; mtDNA: mitochondrial DNA; PC: principal components; PCR: polymerase chain reaction.

## Authors' contributions

KRE designed the research, collected samples, analysed molecular, morphological and ecological data and wrote the manuscript. TKL designed the research, collected samples, conducted most of the analyses for diet and size data and edited the manuscript. AK collected molecular data, analysed molecular data and ran coalescence analyses. CH collected and analysed the stable isotope data and edited the manuscript. AM designed the research, collected samples and helped draft the manuscript.

## Supplementary Material

Additional file 1 **Microsatellite DNA summary statistics**. Microsatellite heterozygosity, the number of alleles and the inbreeding coefficient by locus and lake. Click here for file

Additional file 2 **Factorial correspondence analysis of microsatellite alleles**. Crater lake Apoyeque has extremely reduced genetic variability compared to and is significantly differentiated from, the neighbouring crater lake Xiloá and great lake Managua. Apoyeque (rust; *n *= 386), Xiloá (tan; *n *= 50) and Managua (green; *n *= 185). Click here for file

Additional file 3 **Pairwise population genetic differentiation between lakes**. Matrix of *F*_ST_-values between each population pair: microsatellite differentiation is on the upper right and mitochondrial DNA differentiation is in the lower left of the matrix. Each lake is significantly genetically differentiated from the other. Click here for file

Additional file 4 **Maximal posterior probabilities for three independent iterations of isolation-with-migration coalescence analysis (IMa)**. Maximal posterior probability peaks (HiPt) for three independent runs of IMa. Parameter *q *is the estimated effective population size of (1) Apoyeque, (2) Managua and (a) the ancestral population, in numbers of individuals; *m *is the rate of migration from (1) Managua to Apoyeque and (2) Apoyeque to Managua in average number of migrations per 1 000 generations per gene copy; *t *is the years since divergence between Apoyeque and Managua. Click here for file

Additional file 5 **Specimen list**. Specimens included in the analyses by lake of origin, species, specimen number, lip morph (L = thick-lipped, N = thin-lipped; only relevant for Apoyeque) and sex/maturity (F = adult female, M = adult male, J = juvenile, not noted = not noted or unassignable; only relevant for Apoyeque). Ecological and morphological analyses include: stable isotope analyses (SIA), diet or gut contents (gut), absolute size (body length, head width), and geometric morphometric analyses of body shape and pharyngeal jaw shape. Genetic data include mitochondrial DNA Genbank accession numbers and whether the specimen was genotyped for microsatellite loci (*x *= genotyped). Click here for file

Additional file 6 **Standard body length of Midas cichlids in Apoyeque by lip morphology and sex**. Body size, measured as standard length, did not differ between thin- and thick-lipped individuals (Two factor ANOVA, morph effect, *F*_1,202 _= 0.652, *P *= 0.42). Males were significantly larger than females (sex effect, *F*_1,202 _= 19.9, *P *< 0.001) for both morphs (morph × sex interaction, *F*_1,202 _= 0.082, *P *= 0.78). Click here for file

Additional file 7 **Bivariate analysis of lip size by body length (mm), fitted with quadratic line for thin- and thick-lipped fishes**. A plot of lip size (total lip area, mm^2^) by body length (standard length, mm) with quadratic lines of best-fit shows the different relationship of lip and body size in each of the morphs (*n *= 309 thin-lipped, *n *= 71 thick-lipped). A quadratic (df = 2) had improved goodness-of-fit to the data over a linear (df = 1) regression (thin-lipped: adjusted *R*^2 ^= 0.705 *vs*. 0.672, thick-lipped: adjusted *R*^2 ^0.506 *vs*. 0.461; non-transformed data). Click here for file

Additional file 8 **Frequency distributions of eco-morphological phenotypes and the deviations from normality**. Frequency histograms (left) and normal probability plots (compared to expected proportions under a single normal distribution) (right) for (A) absolute lip size standardized by body length, (B) PC1 of body shape, (B) PC1 of pharyngeal jaw shape, (C) MDS1 for diet inferred from stomach contents and stable isotope values of (D) ^13^C and (E) ^15^N indicative of trophic niche. See Table 2 and text for further information. Click here for file
