# Rapid sympatric ecological differentiation of crater lake cichlid fishes within historic times

**DOI:** 10.1186/1741-7007-10-70

**Published:** 2012-08-07

**Authors:** Kathryn R Elmer, Topi K Lehtonen, Andreas F Kautt, Chris Harrod, Axel Meyer

**Affiliations:** 1 Lehrstuhl fur Zoologie und Evolutionsbiologie, Department of Biology, University of Konstanz, Universitatstrasse 10, 78457 Konstanz, Germany; 2 School of Biological Sciences, Monash University, Victoria 3800, Australia; 3 Department of Evolutionary Genetics, Max Planck Institute for Limnology, Postfach 165, 24302 Plon, Germany; 4 School of Biological Sciences, Queen's University Belfast, Medical Biology Centre, 97 Lisburn Road, Belfast BT9 7BL, UK

## 

The authors noted that the coding and interpretation of Figure five b and five c need corrections [1]. The lines for thin- and thick-lipped fishes' pharyngeal jaws have been reversed (see revised Figure Five b,c (Figure 1 in this article)). After correction, the results for shape differences in lower pharyngeal jaws between fish eco-morphs should instead be interpreted that thin-lipped fishes have a narrower horn, longer jaw and two smaller rear teeth. Therefore, thick-lipped fishes can be generally characterized as more molariform and thin-lipped fishes as more papilliform. These corrections affect statements in: Results page 5, paragraph *Lower pharyngeal jaws*; Discussion page 6, 1st paragraph. We regret the error.

**Figure 1:**
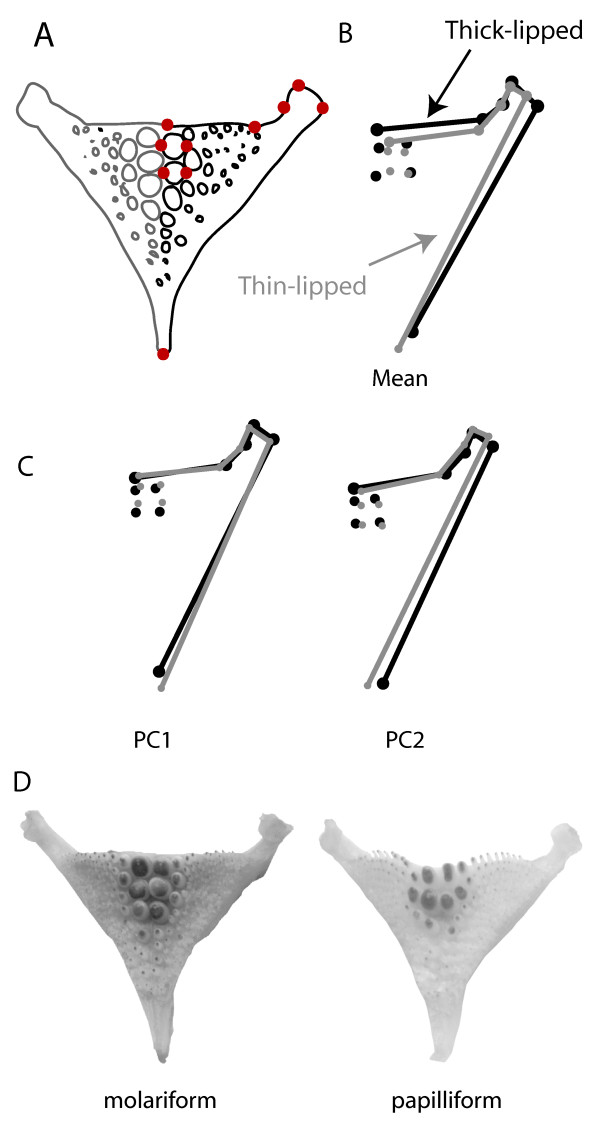
**Revised Figure Five**. Thick-lipped and thin-lipped Midas cichlids in Apoyeque differ in the shape of their pharyngeal jaws. a) Ten homologous landmarks describe jaw shape using one side. b) Discriminant function analysis of mean shape of thick-lipped (black; n = 36) and thin-lipped (grey; n = 135) pharyngeal jaws (scale factor = 4). c) The morphological variation associated with the first two principal component axes (scale factor = 4), responsible for most of the shape variation. d) Exemplars of a molariform and papilliform pharyngeal jaws from Apoyeque Midas cichlids. Note the squatter, broader teeth and thicker horns in the more molariform jaw.

Please note that Topi K Lehtonen can now be contacted at Department of Biology, 20014 University of Turku, Finland. Chris Harrod can now be contacted at Queen's University Belfast, School of Biological Sciences, 97 Lisburn Road, Belfast BT9 7BL, UK and Instituto de Investigaciones Oceanológicas, Universidad de Antofagasta, Avenida Angamos 601, Antofagasta, Chile.
