# Supplementary material for: Rapid sympatric ecological differentiation of crater lake cichlid fishes within historic times
Source: BMC Biol. 2010 May 12;8:60. doi: 10.1186/1741-7007-8-60 (PMC2880021; doi:10.1186/1741-7007-8-60)
Supplement: Additional file 1 — Microsatellite DNA summary statistics. Microsatellite heterozygosity, the number of alleles and the inbreeding coefficient by locus and lake. [file 1741-7007-8-60-S1.pdf]

Additional File 1: Genetic diversity.

Microsatellite heterozygosity, the number of alleles, and the inbreeding coefficient by locus and lake.

**Locus**	**Observed Heterozygosity**	**Expected Heterozygosity**	**Number of Alleles**	F_{IS}
		**Apoyeque**		
Abur82	0.553	0.532	4	-0.040
Abur151	0.719	0.729	6	0.013
M1M	0.374	0.396	3	0.057
M2	0.748	0.724	14	-0.034
M7	0.886	0.858	17	-0.033
TmoM7	0.328	0.349	5	0.059
Burtkit	0.782	0.688	9	-0.137
M12	0.468	0.423	4	-0.107
Unh013	0.776	0.778	14	0.002
Unh002	0.759	0.722	10	-0.052
Unh012	0.563	0.675	8	0.166
mean	0.632	0.625	8.545	-0.010
st.dev	0.176	0.163	4.520	0.080
		**Xiloá**		
Abur82	0.380	0.451	6	0.153
Abur151	0.816	0.784	8	-0.046
M1M	0.327	0.348	3	0.058
M2	0.894	0.876	13	-0.026
M7	0.915	0.894	17	-0.029
TmoM7	0.765	0.705	5	-0.093
Burtkit	0.920	0.837	13	-0.105
M12	0.479	0.567	6	0.151
Unh013	0.721	0.844	11	0.142
Unh002	0.875	0.868	14	-0.013
Unh012	0.905	0.795	9	-0.146
mean	0.727	0.725	9.545	0.004
st.dev	0.214	0.178	4.186	0.102

	Managua			
Abur82	0.619	0.706	8	0.123
Abur151	0.750	0.768	13	0.022
M1M	0.584	0.567	6	-0.030
M2	0.940	0.919	19	-0.025
M7	0.905	0.923	23	0.018
TmoM7	0.488	0.492	9	0.005
Burtkit	0.947	0.914	24	-0.038
M12	0.828	0.828	14	-0.001
Unh013	0.949	0.933	22	-0.018
Unh002	0.819	0.848	20	0.034
Unh012	0.809	0.870	21	0.069
mean	0.785	0.797	16.273	0.014
st.dev	0.152	0.144	6.210	0.046
