# Supplementary figures and images for: Rapid sympatric ecological differentiation of crater lake cichlid fishes within historic times
Source: BMC Biol. 2010 May 12;8:60. doi: 10.1186/1741-7007-8-60 (PMC2880021; doi:10.1186/1741-7007-8-60)

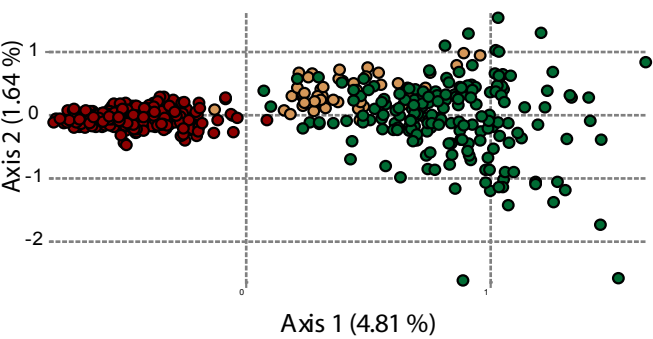

L. Apoyeque ●

L. Xiloá ●

L. Managua ●

Supplement: Additional file 2 — Factorial correspondence analysis of microsatellite alleles. Crater lake Apoyeque has extremely reduced genetic variability compared to and is significantly differentiated from, the neighbouring crater lake Xiloá and great lake Managua. Apoyeque (rust; n = 386), Xiloá (tan; n = 50) and Managua (green; n = 185). [file 1741-7007-8-60-S2.pdf]

A) Lip size

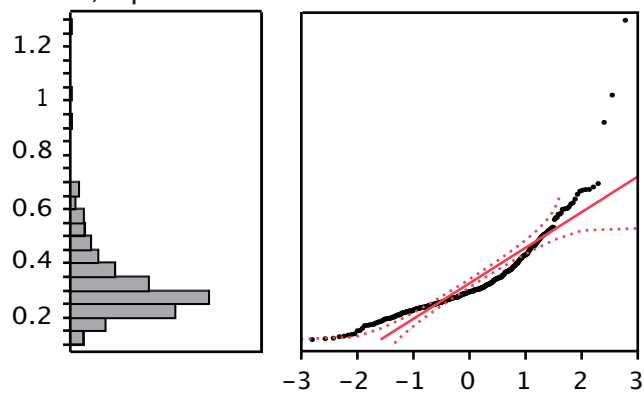

D) Diet

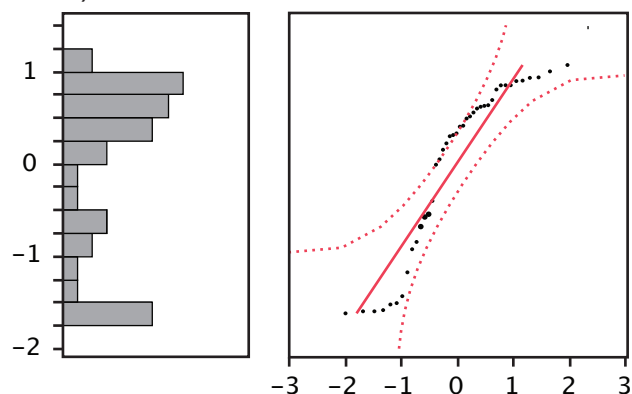

B) Body shape

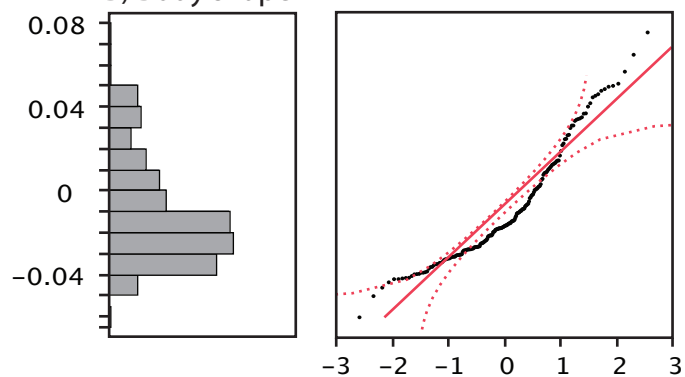E)  $\delta^{13}\text{C}$ 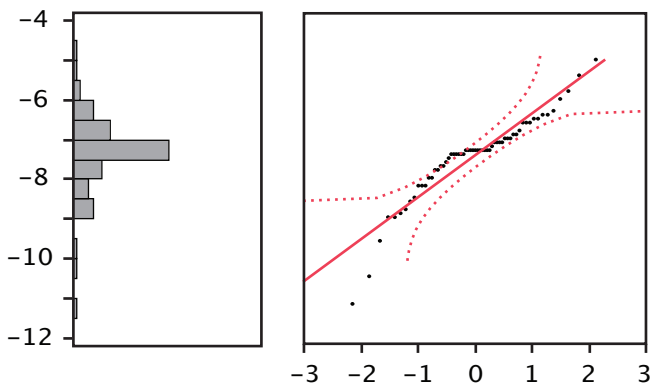

C) Pharyngeal jaws

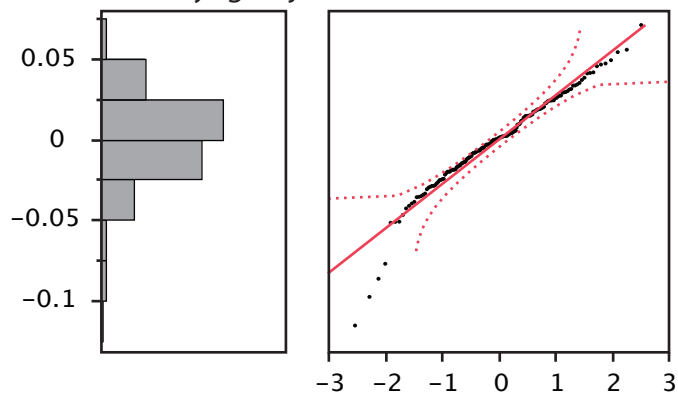F)  $\delta^{15}\text{N}$ 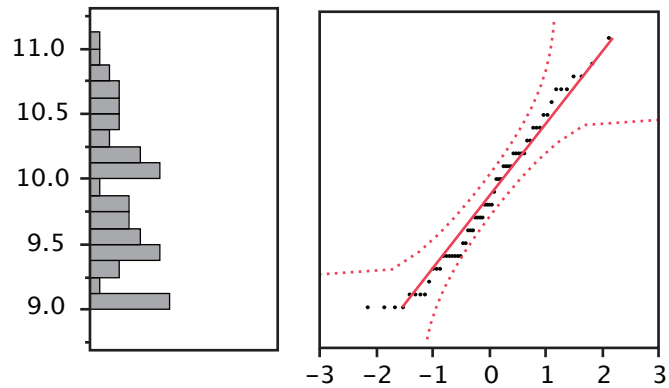

Supplement: Additional file 8 — Frequency distributions of eco-morphological phenotypes and the deviations from normality. Frequency histograms (left) and normal probability plots (compared to expected proportions under a single normal distribution) (right) for (A) absolute lip size standardized by body length, (B) PC1 of body shape, (B) PC1 of pharyngeal jaw shape, (C) MDS1 for diet inferred from stomach contents and stable isotope values of (D) 13C and (E) 15N indicative of trophic niche. See Table 2 and text for further information. [file 1741-7007-8-60-S8.pdf]
