# Supplementary material for: Rapid sympatric ecological differentiation of crater lake cichlid fishes within historic times
Source: BMC Biol. 2010 May 12;8:60. doi: 10.1186/1741-7007-8-60 (PMC2880021; doi:10.1186/1741-7007-8-60)
Supplement: Additional file 3 — Pairwise population genetic differentiation between lakes. Matrix of FST-values between each population pair: microsatellite differentiation is on the upper right and mitochondrial DNA differentiation is in the lower left of the matrix. Each lake is significantly genetically differentiated from the other. [file 1741-7007-8-60-S3.pdf]

Additional File 3: Population differentiation across lakes.

Matrix of F_{ST}-values between each population pair: microsatellite differentiation is on the upper right and mtDNA differentiation is in the lower left of the matrix. Each lake is significantly genetically differentiated from the other.

	**Apoyeque**	**Xiloá**	**Managua**
**Apoyeque**	-	0.141**	0.146**
**Xiloá**	0.125*	-	0.053**
**Managua**	0.262**	0.072*	-

(** p < 0.001, * p < 0.01). Results are the same if populations are compared the other way around.
