# Supplementary material for: Rapid sympatric ecological differentiation of crater lake cichlid fishes within historic times
Source: BMC Biol. 2010 May 12;8:60. doi: 10.1186/1741-7007-8-60 (PMC2880021; doi:10.1186/1741-7007-8-60)
Supplement: Additional file 4 — Maximal posterior probabilities for three independent iterations of isolation-with-migration coalescence analysis (IMa). Maximal posterior probability peaks (HiPt) for three independent runs of IMa. Parameter q is the estimated effective population size of (1) Apoyeque, (2) Managua and (a) the ancestral population, in numbers of individuals; m is the rate of migration from (1) Managua to Apoyeque and (2) Apoyeque to Managua in average number of migrations per 1 000 generations per gene copy; t is the years since divergence between Apoyeque and Managua. [file 1741-7007-8-60-S4.pdf]

Additional File 4: Maximal posterior probability peaks (HiPt) for three independent runs of IMA.

Parameter *q* is the estimated effective population size of (1) Apoyeque, (2) Managua, and (a) the ancestral population, in numbers of individuals; *m* is the rate of migration from (1) Managua to Apoyeque and (2) Apoyeque to Managua in average number of migrations per 1000 generations per gene copy; *t* is the years since divergence between Apoyeque and Managua.

	***q*1**	***q*2**	***q*a**	***m*1**	***m*2**	***t***
Run 1	28	243	36108	8	0.2	89
Run 2	39	2435	27258	12	0.3	63
Run 3	7	290	29109	12	0.2	132
