# Supplementary material for: Rapid sympatric ecological differentiation of crater lake cichlid fishes within historic times
Source: BMC Biol. 2010 May 12;8:60. doi: 10.1186/1741-7007-8-60 (PMC2880021; doi:10.1186/1741-7007-8-60)
Supplement: Additional file 5 — Specimen list. Specimens included in the analyses by lake of origin, species, specimen number, lip morph (L = thick-lipped, N = thin-lipped; only relevant for Apoyeque) and sex/maturity (F = adult female, M = adult male, J = juvenile, not noted = not noted or unassignable; only relevant for Apoyeque). Ecological and morphological analyses include: stable isotope analyses (SIA), diet or gut contents (gut), absolute size (body length, head width), and geometric morphometric analyses of body shape and pharyngeal jaw shape. Genetic data include mitochondrial DNA Genbank accession numbers and whether the specimen was genotyped for microsatellite loci (x = genotyped). [file 1741-7007-8-60-S5.pdf]

**Additional File 5: Specimen list**

Specimens included in the analyses by lake of origin, species, specimen number, lip morph (L = thick-lipped, N = thin-lipped; only relevant for Apoyeque), and sex/maturity (F = adult female, M = adult male, J = juvenile, not noted = not noted or unassignable; only relevant for Apoyeque). Ecological and morphological analyses include: stable isotope analyses (SIA), diet or gut contents (gut), head width, lip and body size, and geometric morphometric analyses of body shape and pharyngeal jaw shape. Genetic data include mtDNA Genbank accession numbers, and whether the specimen was genotyped for microsatellite loci (x = genotyped).

| Lake | Species | sample ID | lip | sex/ maturity | SIA | diet | width | shape | shape | lip & body size | Genbank | Genotype |
|---|---|---|---|---|---|---|---|---|---|---|---|---|
| Apoyeque | *A.* cf. *citrinellus* | AMCK00378 | L | M | SIA | gut | | | | x | | x |
| Apoyeque | *A.* cf. *citrinellus* | AMCK00379 | N | not noted | | gut | | | | x | GU016704 | x |
| Apoyeque | *A.* cf. *citrinellus* | AMCK00380 | N | not noted | | | | | | x | GU016705 | x |
| Apoyeque | *A.* cf. *citrinellus* | AMCK00381 | N | F | SIA | gut | head | body | jaw | x | GU016706 | x |
| Apoyeque | *A.* cf. *citrinellus* | AMCK00382 | L | not noted | SIA | gut | | | | x | GU016707 | x |
| Apoyeque | *A.* cf. *citrinellus* | AMCK00383 | L | not noted | SIA | gut | | | | x | GU016708 | x |
| Apoyeque | *A.* cf. *citrinellus* | AMCK00384 | L | F | SIA | gut | head | body | jaw | x | GU016709 | x |
| Apoyeque | *A.* cf. *citrinellus* | AMCK00385 | L | not noted | SIA | gut | | | | x | GU016710 | x |
| Apoyeque | *A.* cf. *citrinellus* | AMCK00386 | N | not noted | SIA | gut | | | | x | GU016711 | x |
| Apoyeque | *A.* cf. *citrinellus* | AMCK00387 | N | not noted | SIA | gut | | | | x | GU016712 | |
| Apoyeque | *A.* cf. *citrinellus* | AMCK00388 | N | not noted | SIA | | | | | x | GU016713 | x |
| Apoyeque | *A.* cf. *citrinellus* | AMCK00389 | N | not noted | | | | | | x | GU016714 | x |
| Apoyeque | *A.* cf. *citrinellus* | AMCK00390 | N | M | | | | | | x | GU016715 | x |
| Apoyeque | *A.* cf. *citrinellus* | AMCK00391 | N | not noted | | | | | | x | | x |
| Apoyeque | *A.* cf. *citrinellus* | AMCK00392 | N | not noted | | | | | | x | | x |

| | | | | | | | | | | | | |
|---|---|---|---|---|---|---|---|---|---|---|---|---|
| Apoyeque | *A.* cf. *citrinellus* | AMCK00393 | N | F | SIA | | head | body | jaw | x | GU016716 | x |
| Apoyeque | *A.* cf. *citrinellus* | AMCK00394 | N | not noted | | | | | | x | GU016717 | x |
| Apoyeque | *A.* cf. *citrinellus* | AMCK00395 | N | M | SIA | gut | head | body | jaw | x | GU016718 | x |
| Apoyeque | *A.* cf. *citrinellus* | AMCK00396 | N | M | SIA | | head | body | jaw | x | GU016719 | x |
| Apoyeque | *A.* cf. *citrinellus* | AMCK00397 | N | F | SIA | gut | head | body | jaw | | GU016720 | x |
| Apoyeque | *A.* cf. *citrinellus* | AMCK00398 | N | M | SIA | | head | body | jaw | x | GU016721 | x |
| Apoyeque | *A.* cf. *citrinellus* | AMCK00399 | N | not noted | | | | | | x | GU016722 | x |
| Apoyeque | *A.* cf. *citrinellus* | AMCK00400 | N | not noted | | | | | | x | GU016723 | x |
| Apoyeque | *A.* cf. *citrinellus* | AMCK00401 | N | F | SIA | gut | head | body | jaw | x | GU016724 | x |
| Apoyeque | *A.* cf. *citrinellus* | AMCK00402 | N | not noted | | | | | | x | GU016725 | x |
| Apoyeque | *A.* cf. *citrinellus* | AMCK00403 | N | F | SIA | | | | | x | GU016726 | x |
| Apoyeque | *A.* cf. *citrinellus* | AMCK00404 | N | not noted | | | | | | | GU016727 | x |
| Apoyeque | *A.* cf. *citrinellus* | AMCK00405 | N | not noted | | | | | | x | GU016728 | x |
| Apoyeque | *A.* cf. *citrinellus* | AMCK00406 | N | not noted | | | | | | x | GU016729 | x |
| Apoyeque | *A.* cf. *citrinellus* | AMCK00407 | N | not noted | | | | | | x | GU016730 | x |
| Apoyeque | *A.* cf. *citrinellus* | AMCK00408 | N | not noted | | | | | | x | GU016731 | x |
| Apoyeque | *A.* cf. *citrinellus* | AMCK00409 | N | not noted | | | | | | x | GU016732 | x |
| Apoyeque | *A.* cf. *citrinellus* | AMCK00410 | N | not noted | | | | | | x | GU016733 | x |
| Apoyeque | *A.* cf. *citrinellus* | AMCK00411 | N | not noted | | | | | | x | GU016734 | x |
| Apoyeque | *A.* cf. *citrinellus* | AMCK00412 | N | not noted | | | | | | x | GU016735 | x |
| Apoyeque | *A.* cf. *citrinellus* | AMCK00413 | N | not noted | | | | | | x | GU016736 | x |
| Apoyeque | *A.* cf. *citrinellus* | AMCK00414 | N | not noted | | | | | | x | GU016737 | x |
| Apoyeque | *A.* cf. *citrinellus* | AMCK00415 | N | not noted | | | | | | x | GU016738 | x |
| Apoyeque | *A.* cf. *citrinellus* | AMCK00416 | L | not noted | | | | | | x | GU016739 | x |
| Apoyeque | *A.* cf. *citrinellus* | AMCK00417 | N | not noted | | | | | | x | GU016740 | x |
| Apoyeque | *A.* cf. *citrinellus* | AMCK00418 | N | not noted | | | | | | x | GU016741 | x |
| Apoyeque | *A.* cf. *citrinellus* | AMCK00419 | N | not noted | SIA | gut | | | | x | GU016742 | x |
| Apoyeque | *A.* cf. *citrinellus* | AMCK00420 | N | not noted | SIA | gut | | | | x | GU016743 | x |
| Apoyeque | *A.* cf. *citrinellus* | AMCK00421 | N | not noted | SIA | | | | | x | GU016744 | x |
| Apoyeque | *A.* cf. *citrinellus* | AMCK00422 | N | not noted | | | | | | x | GU016745 | x |

| | | | | | | | | |
|---|---|---|---|---|---|---|---|---|
| Apoyeque | *A.* cf. *citrinellus* | AMCK00423 | N | not noted | | x | GU016746 | x |
| Apoyeque | *A.* cf. *citrinellus* | AMCK00424 | N | not noted | | x | GU016747 | x |
| Apoyeque | *A.* cf. *citrinellus* | AMCK00425 | N | not noted | | x | GU016748 | x |
| Apoyeque | *A.* cf. *citrinellus* | AMCK00426 | N | not noted | | x | GU016749 | x |
| Apoyeque | *A.* cf. *citrinellus* | AMCK00427 | N | not noted | SIA | x | GU016750 | x |
| Apoyeque | *A.* cf. *citrinellus* | AMCK00428 | N | not noted | | x | GU016751 | x |
| Apoyeque | *A.* cf. *citrinellus* | AMCK00429 | N | not noted | | x | GU016752 | x |
| Apoyeque | *A.* cf. *citrinellus* | AMCK00430 | N | not noted | | x | GU016753 | x |
| Apoyeque | *A.* cf. *citrinellus* | AMCK00431 | N | not noted | | x | GU016754 | x |
| Apoyeque | *A.* cf. *citrinellus* | AMCK00432 | N | not noted | | x | GU016755 | x |
| Apoyeque | *A.* cf. *citrinellus* | AMCK00433 | N | not noted | | x | GU016756 | x |
| Apoyeque | *A.* cf. *citrinellus* | AMCK00434 | N | not noted | | x | GU016757 | x |
| Apoyeque | *A.* cf. *citrinellus* | AMCK00435 | N | not noted | | x | GU016758 | x |
| Apoyeque | *A.* cf. *citrinellus* | AMCK00436 | N | not noted | | x | GU016759 | x |
| Apoyeque | *A.* cf. *citrinellus* | AMCK00437 | N | not noted | | x | GU016760 | x |
| Apoyeque | *A.* cf. *citrinellus* | AMCK00438 | N | not noted | | x | GU016761 | x |
| Apoyeque | *A.* cf. *citrinellus* | AMCK00439 | N | not noted | | x | GU016762 | x |
| Apoyeque | *A.* cf. *citrinellus* | AMCK00440 | N | not noted | | x | GU016763 | x |
| Apoyeque | *A.* cf. *citrinellus* | AMCK00441 | N | not noted | | x | GU016764 | x |
| Apoyeque | *A.* cf. *citrinellus* | AMCK00442 | N | not noted | | x | GU016765 | x |
| Apoyeque | *A.* cf. *citrinellus* | AMCK00443 | N | not noted | | x | GU016766 | x |
| Apoyeque | *A.* cf. *citrinellus* | AMCK00444 | N | not noted | | x | GU016767 | x |
| Apoyeque | *A.* cf. *citrinellus* | AMCK00445 | N | not noted | | x | GU016768 | x |
| Apoyeque | *A.* cf. *citrinellus* | AMCK00446 | N | not noted | | x | GU016769 | x |
| Apoyeque | *A.* cf. *citrinellus* | AMCK00447 | N | not noted | | x | GU016770 | x |
| Apoyeque | *A.* cf. *citrinellus* | AMCK00448 | L | not noted | | x | GU016771 | x |
| Apoyeque | *A.* cf. *citrinellus* | AMCK00449 | N | not noted | | x | GU016772 | x |
| Apoyeque | *A.* cf. *citrinellus* | AMCK00450 | L | not noted | | x | GU016773 | x |
| Apoyeque | *A.* cf. *citrinellus* | AMCK00451 | N | not noted | | x | GU016774 | x |
| Apoyeque | *A.* cf. *citrinellus* | AMCK00452 | N | not noted | | x | GU016775 | x |

| | | | | | | | | | | | |
|---|---|---|---|---|---|---|---|---|---|---|---|
| Apoyeque | *A.* cf. *citrinellus* | AMCK00453 | N | not noted | | | | | x | GU016776 | x |
| Apoyeque | *A.* cf. *citrinellus* | AMCK00454 | N | not noted | | | | | x | GU016777 | x |
| Apoyeque | *A.* cf. *citrinellus* | AMCK00455 | N | not noted | | | | | x | GU016778 | x |
| Apoyeque | *A.* cf. *citrinellus* | AMCK00456 | N | not noted | | | | | x | GU016779 | x |
| Apoyeque | *A.* cf. *citrinellus* | AMCK00457 | N | F | SIA | | | | x | GU016780 | x |
| Apoyeque | *A.* cf. *citrinellus* | AMCK00458 | N | not noted | | | | | x | GU016781 | x |
| Apoyeque | *A.* cf. *citrinellus* | AMCK00580 | N | J | | | | | | GU016782 | x |
| Apoyeque | *A.* cf. *citrinellus* | AMCK00644 | N | M | | head | body | jaw | x | GU016783 | x |
| Apoyeque | *A.* cf. *citrinellus* | AMCK00645 | N | M | | head | body | jaw | x | GU016784 | x |
| Apoyeque | *A.* cf. *citrinellus* | AMCK00646 | L | J | | | | | x | GU016785 | x |
| Apoyeque | *A.* cf. *citrinellus* | AMCK00647 | N | not noted | | | | | x | GU016786 | x |
| Apoyeque | *A.* cf. *citrinellus* | AMCK00648 | L | M | SIA | head | body | jaw | x | GU016787 | x |
| Apoyeque | *A.* cf. *citrinellus* | AMCK00649 | N | J | | | | | x | GU016788 | x |
| Apoyeque | *A.* cf. *citrinellus* | AMCK00650 | N | F | | head | body | jaw | x | GU016789 | x |
| Apoyeque | *A.* cf. *citrinellus* | AMCK00651 | N | M | | head | body | jaw | x | | |
| Apoyeque | *A.* cf. *citrinellus* | AMCK00652 | L | not noted | SIA | | | | x | | |
| Apoyeque | *A.* cf. *citrinellus* | AMCK00653 | N | M | | head | body | jaw | x | | x |
| Apoyeque | *A.* cf. *citrinellus* | AMCK00654 | N | F | | head | body | jaw | x | GU016790 | x |
| Apoyeque | *A.* cf. *citrinellus* | AMCK00655 | N | F | | head | body | jaw | x | | |
| Apoyeque | *A.* cf. *citrinellus* | AMCK00656 | N | F | | head | body | jaw | x | GU016791 | x |
| Apoyeque | *A.* cf. *citrinellus* | AMCK00657 | N | M | | head | body | jaw | x | | x |
| Apoyeque | *A.* cf. *citrinellus* | AMCK00658 | N | F | | head | body | jaw | x | | x |
| Apoyeque | *A.* cf. *citrinellus* | AMCK00659 | N | F | | head | body | jaw | x | | |
| Apoyeque | *A.* cf. *citrinellus* | AMCK00660 | N | F | | head | body | jaw | x | | x |
| Apoyeque | *A.* cf. *citrinellus* | AMCK00661 | N | M | | head | body | jaw | x | | x |
| Apoyeque | *A.* cf. *citrinellus* | AMCK00662 | L | M | | head | body | jaw | x | | |
| Apoyeque | *A.* cf. *citrinellus* | AMCK00663 | N | M | | head | body | jaw | x | | |
| Apoyeque | *A.* cf. *citrinellus* | AMCK00664 | L | M | SIA | head | body | jaw | x | | x |
| Apoyeque | *A.* cf. *citrinellus* | AMCK00665 | N | not noted | | | | | x | | x |
| Apoyeque | *A.* cf. *citrinellus* | AMCK00666 | N | J | | | | | x | | x |
| Apoyeque | *A.* cf. *citrinellus* | AMCK00667 | N | F | | head | body | jaw | x | | x |

| | | | | | | | | | | | | |
|---|---|---|---|---|---|---|---|---|---|---|---|---|
| Apoyeque | *A.* cf. *citrinellus* | AMCK00668 | N | M | | | head | body | jaw | x | | x |
| Apoyeque | *A.* cf. *citrinellus* | AMCK00669 | N | M | | | head | body | jaw | x | | x |
| Apoyeque | *A.* cf. *citrinellus* | AMCK00670 | N | not noted | | | | | | x | | |
| Apoyeque | *A.* cf. *citrinellus* | AMCK00671 | N | M | | | head | body | jaw | x | | x |
| Apoyeque | *A.* cf. *citrinellus* | AMCK00672 | N | F | | | head | body | jaw | x | | x |
| Apoyeque | *A.* cf. *citrinellus* | AMCK00673 | N | F | | | head | body | jaw | x | | x |
| Apoyeque | *A.* cf. *citrinellus* | AMCK00674 | N | M | | | head | body | jaw | x | | x |
| Apoyeque | *A.* cf. *citrinellus* | AMCK00675 | N | M | | | head | body | jaw | x | | x |
| Apoyeque | *A.* cf. *citrinellus* | AMCK00676 | N | not noted | | | | | | x | GU016792 | x |
| Apoyeque | *A.* cf. *citrinellus* | AMCK00677 | N | not noted | | | | | | x | | x |
| Apoyeque | *A.* cf. *citrinellus* | AMCK00678 | L | not noted | SIA | | | | | x | GU016793 | x |
| Apoyeque | *A.* cf. *citrinellus* | AMCK00679 | N | F | | | | | | x | | x |
| Apoyeque | *A.* cf. *citrinellus* | AMCK00680 | N | J | | | | | | x | | x |
| Apoyeque | *A.* cf. *citrinellus* | AMCK00681 | L | J | | | | | | x | | x |
| Apoyeque | *A.* cf. *citrinellus* | AMCK00682 | L | F | | | head | body | jaw | x | | x |
| Apoyeque | *A.* cf. *citrinellus* | AMCK00683 | N | M | | | head | body | jaw | x | | x |
| Apoyeque | *A.* cf. *citrinellus* | AMCK00684 | N | F | | | head | body | jaw | x | | x |
| Apoyeque | *A.* cf. *citrinellus* | AMCK00685 | N | F | SIA | gut | | | | x | | x |
| Apoyeque | *A.* cf. *citrinellus* | AMCK00686 | N | M | SIA | gut | head | body | jaw | x | | x |
| Apoyeque | *A.* cf. *citrinellus* | AMCK00687 | N | M | | | head | body | jaw | x | | x |
| Apoyeque | *A.* cf. *citrinellus* | AMCK00688 | N | J | | | | | | x | GU016794 | x |
| Apoyeque | *A.* cf. *citrinellus* | AMCK00689 | L | M | SIA | | head | body | jaw | x | GU016795 | x |
| Apoyeque | *A.* cf. *citrinellus* | AMCK00690 | N | F | SIA | | head | body | jaw | x | | x |
| Apoyeque | *A.* cf. *citrinellus* | AMCK00691 | N | M | | | head | body | jaw | x | GU016796 | x |
| Apoyeque | *A.* cf. *citrinellus* | AMCK00692 | N | M | | | | | | x | | x |
| Apoyeque | *A.* cf. *citrinellus* | AMCK00693 | N | F | | | head | body | jaw | x | GU016797 | x |
| Apoyeque | *A.* cf. *citrinellus* | AMCK00694 | N | J | | | | | | x | GU016798 | x |
| Apoyeque | *A.* cf. *citrinellus* | AMCK00695 | N | M | | | | | | x | | x |
| Apoyeque | *A.* cf. *citrinellus* | AMCK00696 | N | F | | | | | | x | | x |
| Apoyeque | *A.* cf. *citrinellus* | AMCK00697 | N | M | | | head | body | jaw | x | | x |
| Apoyeque | *A.* cf. *citrinellus* | AMCK00698 | L | M | | | head | body | jaw | x | | x |

| | | | | | | | | | | | |
|---|---|---|---|---|---|---|---|---|---|---|---|
| Apoyeque | *A.* cf. *citrinellus* | AMCK00699 | N | M | | head | body | jaw | x | | x |
| Apoyeque | *A.* cf. *citrinellus* | AMCK00700 | N | F | | head | body | jaw | x | | x |
| Apoyeque | *A.* cf. *citrinellus* | AMCK00701 | N | J | | | | | x | | x |
| Apoyeque | *A.* cf. *citrinellus* | AMCK00702 | L | J | | | | | x | | x |
| Apoyeque | *A.* cf. *citrinellus* | AMCK00703 | N | J | | | | | | | x |
| Apoyeque | *A.* cf. *citrinellus* | AMCK00704 | N | F | | head | body | jaw | x | | x |
| Apoyeque | *A.* cf. *citrinellus* | AMCK00705 | N | M | | head | body | jaw | x | | x |
| Apoyeque | *A.* cf. *citrinellus* | AMCK00706 | N | not noted | | | | | x | | x |
| Apoyeque | *A.* cf. *citrinellus* | AMCK00707 | N | F | | head | body | jaw | x | | x |
| Apoyeque | *A.* cf. *citrinellus* | AMCK00708 | L | F | | head | body | jaw | x | | x |
| Apoyeque | *A.* cf. *citrinellus* | AMCK00709 | N | J | | | | | x | | x |
| Apoyeque | *A.* cf. *citrinellus* | AMCK00710 | L | J | | | | | x | | x |
| Apoyeque | *A.* cf. *citrinellus* | AMCK00711 | L | F | | head | body | jaw | x | | x |
| Apoyeque | *A.* cf. *citrinellus* | AMCK00712 | N | F | | head | body | jaw | x | | x |
| Apoyeque | *A.* cf. *citrinellus* | AMCK00713 | N | J | | | | | x | | x |
| Apoyeque | *A.* cf. *citrinellus* | AMCK00714 | N | F | | head | body | jaw | x | | x |
| Apoyeque | *A.* cf. *citrinellus* | AMCK00715 | L | M | SIA | head | body | jaw | x | | x |
| Apoyeque | *A.* cf. *citrinellus* | AMCK00716 | N | F | | head | body | jaw | x | | x |
| Apoyeque | *A.* cf. *citrinellus* | AMCK00717 | N | J | | | | | x | | x |
| Apoyeque | *A.* cf. *citrinellus* | AMCK00718 | L | M | | head | body | jaw | x | | x |
| Apoyeque | *A.* cf. *citrinellus* | AMCK00719 | N | M | | | | | x | | |
| Apoyeque | *A.* cf. *citrinellus* | AMCK00720 | N | F | | head | body | jaw | x | | x |
| Apoyeque | *A.* cf. *citrinellus* | AMCK00721 | N | M | | head | body | jaw | x | | x |
| Apoyeque | *A.* cf. *citrinellus* | AMCK00722 | N | not noted | | | | | x | GU016799 | x |
| Apoyeque | *A.* cf. *citrinellus* | AMCK00723 | N | F | | head | body | jaw | x | | x |
| Apoyeque | *A.* cf. *citrinellus* | AMCK00724 | N | J | | | | | x | | x |
| Apoyeque | *A.* cf. *citrinellus* | AMCK00725 | N | J | | | | | x | | x |
| Apoyeque | *A.* cf. *citrinellus* | AMCK00726 | N | F | | head | body | jaw | x | | x |
| Apoyeque | *A.* cf. *citrinellus* | AMCK00727 | N | M | | head | body | jaw | x | | x |
| Apoyeque | *A.* cf. *citrinellus* | AMCK00728 | N | J | | | | | x | | x |

| | | | | | | | | | | | |
|---|---|---|---|---|---|---|---|---|---|---|---|
| Apoyeque | *A.* cf. *citrinellus* | AMCK00729 | N | F | | head | body | jaw | x | | x |
| Apoyeque | *A.* cf. *citrinellus* | AMCK00730 | N | F | | | | | x | | x |
| Apoyeque | *A.* cf. *citrinellus* | AMCK00731 | N | J | | | | | x | | x |
| Apoyeque | *A.* cf. *citrinellus* | AMCK00732 | N | M | | head | body | jaw | x | | x |
| Apoyeque | *A.* cf. *citrinellus* | AMCK00733 | N | F | | | | | x | | x |
| Apoyeque | *A.* cf. *citrinellus* | AMCK00734 | L | F | | | | | x | | x |
| Apoyeque | *A.* cf. *citrinellus* | AMCK00735 | N | J | | | | | x | | x |
| Apoyeque | *A.* cf. *citrinellus* | AMCK00736 | N | J | | | | | x | | x |
| Apoyeque | *A.* cf. *citrinellus* | AMCK00737 | N | F | | head | body | jaw | x | | x |
| Apoyeque | *A.* cf. *citrinellus* | AMCK00738 | N | M | | head | body | jaw | x | | x |
| Apoyeque | *A.* cf. *citrinellus* | AMCK00739 | N | F | | head | body | jaw | x | | x |
| Apoyeque | *A.* cf. *citrinellus* | AMCK00740 | L | M | SIA | head | body | jaw | x | | x |
| Apoyeque | *A.* cf. *citrinellus* | AMCK00741 | N | M | | head | body | jaw | x | | x |
| Apoyeque | *A.* cf. *citrinellus* | AMCK00742 | N | M | | | | | x | | x |
| Apoyeque | *A.* cf. *citrinellus* | AMCK00743 | N | F | | head | body | jaw | x | | x |
| Apoyeque | *A.* cf. *citrinellus* | AMCK00744 | N | F | | | | | x | | x |
| Apoyeque | *A.* cf. *citrinellus* | AMCK00745 | N | M | | head | body | jaw | x | | x |
| Apoyeque | *A.* cf. *citrinellus* | AMCK00746 | N | M | | head | body | jaw | x | | x |
| Apoyeque | *A.* cf. *citrinellus* | AMCK00747 | N | not noted | | | | | x | | x |
| Apoyeque | *A.* cf. *citrinellus* | AMCK00748 | N | F | | head | body | jaw | x | | x |
| Apoyeque | *A.* cf. *citrinellus* | AMCK00749 | N | not noted | | | | | x | | x |
| Apoyeque | *A.* cf. *citrinellus* | AMCK00750 | N | M | | head | body | jaw | x | | x |
| Apoyeque | *A.* cf. *citrinellus* | AMCK00751 | L | J | | | | | x | | x |
| Apoyeque | *A.* cf. *citrinellus* | AMCK00752 | N | F | | head | body | jaw | x | | x |
| Apoyeque | *A.* cf. *citrinellus* | AMCK00753 | N | J | | | | | x | | x |
| Apoyeque | *A.* cf. *citrinellus* | AMCK00754 | N | M | | head | body | jaw | x | | x |
| Apoyeque | *A.* cf. *citrinellus* | AMCK00755 | L | F | | head | body | jaw | x | GU016800 | x |
| Apoyeque | *A.* cf. *citrinellus* | AMCK00756 | N | J | | | | | x | GU016801 | x |
| Apoyeque | *A.* cf. *citrinellus* | AMCK00757 | N | M | | head | body | jaw | x | GU016802 | x |
| Apoyeque | *A.* cf. *citrinellus* | AMCK00758 | N | F | | head | body | jaw | x | GU016803 | x |

| | | | | | | | | | | | |
|---|---|---|---|---|---|---|---|---|---|---|---|
| Apoyeque | *A.* cf. *citrinellus* | AMCK00759 | N | F | | | | | x | GU016804 | x |
| Apoyeque | *A.* cf. *citrinellus* | AMCK00760 | L | J | | | | | x | GU016805 | x |
| Apoyeque | *A.* cf. *citrinellus* | AMCK00761 | L | J | | | | | x | GU016806 | x |
| Apoyeque | *A.* cf. *citrinellus* | AMCK00762 | N | F | | head | body | jaw | x | GU016807 | x |
| Apoyeque | *A.* cf. *citrinellus* | AMCK00763 | N | F | | head | body | jaw | x | GU016808 | x |
| Apoyeque | *A.* cf. *citrinellus* | AMCK00764 | N | J | | | | | x | GU016809 | x |
| Apoyeque | *A.* cf. *citrinellus* | AMCK00765 | L | J | | | | | x | GU016810 | x |
| Apoyeque | *A.* cf. *citrinellus* | AMCK00766 | N | J | | | | | x | GU016811 | x |
| Apoyeque | *A.* cf. *citrinellus* | AMCK00767 | N | M | | head | body | jaw | x | GU016812 | x |
| Apoyeque | *A.* cf. *citrinellus* | AMCK00768 | N | J | | | | | x | GU016813 | x |
| Apoyeque | *A.* cf. *citrinellus* | AMCK00769 | N | not noted | | | | | x | GU016814 | x |
| Apoyeque | *A.* cf. *citrinellus* | AMCK00770 | L | J | | | | | x | GU016815 | x |
| Apoyeque | *A.* cf. *citrinellus* | AMCK00771 | N | J | | | | | x | GU016816 | x |
| Apoyeque | *A.* cf. *citrinellus* | AMCK00772 | N | J | | | | | x | | x |
| Apoyeque | *A.* cf. *citrinellus* | AMCK00773 | N | J | | | | | x | GU016817 | x |
| Apoyeque | *A.* cf. *citrinellus* | AMCK00774 | N | F | | | | | x | GU016818 | x |
| Apoyeque | *A.* cf. *citrinellus* | AMCK00775 | L | F | | head | body | jaw | x | GU016819 | x |
| Apoyeque | *A.* cf. *citrinellus* | AMCK00776 | L | J | | | | | x | GU016820 | x |
| Apoyeque | *A.* cf. *citrinellus* | AMCK00777 | N | M | | | | | x | GU016821 | x |
| Apoyeque | *A.* cf. *citrinellus* | AMCK00778 | N | J | | | | | x | GU016822 | x |
| Apoyeque | *A.* cf. *citrinellus* | AMCK00779 | N | M | | | | | x | GU016823 | x |
| Apoyeque | *A.* cf. *citrinellus* | AMCK00780 | N | F | | | | | x | GU016824 | x |
| Apoyeque | *A.* cf. *citrinellus* | AMCK00781 | L | J | | | | | x | GU016825 | x |
| Apoyeque | *A.* cf. *citrinellus* | AMCK00782 | L | M | SIA | head | body | jaw | x | GU016826 | x |
| Apoyeque | *A.* cf. *citrinellus* | AMCK00783 | N | J | | | | | x | GU016827 | x |
| Apoyeque | *A.* cf. *citrinellus* | AMCK00784 | L | J | | | | | x | GU016828 | x |
| Apoyeque | *A.* cf. *citrinellus* | AMCK00785 | N | F | | head | body | jaw | x | GU016829 | x |
| Apoyeque | *A.* cf. *citrinellus* | AMCK00786 | N | J | | | | | x | GU016830 | x |
| Apoyeque | *A.* cf. *citrinellus* | AMCK00787 | N | J | | | | | x | GU016831 | x |
| Apoyeque | *A.* cf. *citrinellus* | AMCK00788 | N | J | | | | | x | GU016832 | x |

| | | | | | | | | | | | |
|---|---|---|---|---|---|---|---|---|---|---|---|
| Apoyeque | *A.* cf. *citrinellus* | AMCK00789 | N | M | | | | | x | GU016833 | x |
| Apoyeque | *A.* cf. *citrinellus* | AMCK00790 | N | M | | | | | x | GU016834 | x |
| Apoyeque | *A.* cf. *citrinellus* | AMCK00791 | N | not noted | | | | | x | GU016835 | x |
| Apoyeque | *A.* cf. *citrinellus* | AMCK00792 | N | not noted | | | | | x | GU016836 | x |
| Apoyeque | *A.* cf. *citrinellus* | AMCK00793 | N | not noted | | | | | x | GU016837 | x |
| Apoyeque | *A.* cf. *citrinellus* | AMCK00794 | N | J | | | | | x | GU016838 | x |
| Apoyeque | *A.* cf. *citrinellus* | AMCK00795 | L | F | SIA | head | body | jaw | x | GU016839 | x |
| Apoyeque | *A.* cf. *citrinellus* | AMCK00796 | L | J | | | | | x | GU016840 | x |
| Apoyeque | *A.* cf. *citrinellus* | AMCK00797 | N | F | | head | body | jaw | x | GU016841 | x |
| Apoyeque | *A.* cf. *citrinellus* | AMCK00798 | N | M | | | | | x | GU016842 | x |
| Apoyeque | *A.* cf. *citrinellus* | AMCK00799 | L | M | SIA | head | body | jaw | x | GU016843 | x |
| Apoyeque | *A.* cf. *citrinellus* | AMCK00800 | N | F | | head | body | jaw | x | GU016844 | x |
| Apoyeque | *A.* cf. *citrinellus* | AMCK00801 | N | J | | | | | x | GU016845 | x |
| Apoyeque | *A.* cf. *citrinellus* | AMCK00802 | N | F | | head | body | jaw | x | GU016846 | x |
| Apoyeque | *A.* cf. *citrinellus* | AMCK00803 | L | M | SIA | head | body | jaw | x | GU016847 | x |
| Apoyeque | *A.* cf. *citrinellus* | AMCK00804 | N | F | | head | body | jaw | x | GU016848 | x |
| Apoyeque | *A.* cf. *citrinellus* | AMCK00805 | L | F | SIA | head | body | jaw | x | GU016849 | x |
| Apoyeque | *A.* cf. *citrinellus* | AMCK00806 | L | F | | head | body | jaw | x | GU016850 | x |
| Apoyeque | *A.* cf. *citrinellus* | AMCK00807 | N | F | | | | | x | GU016851 | x |
| Apoyeque | *A.* cf. *citrinellus* | AMCK00808 | N | J | | | | | x | GU016852 | x |
| Apoyeque | *A.* cf. *citrinellus* | AMCK00809 | N | J | | | | | x | GU016853 | x |
| Apoyeque | *A.* cf. *citrinellus* | AMCK00810 | L | J | | | | | x | GU016854 | x |
| Apoyeque | *A.* cf. *citrinellus* | AMCK00811 | N | F | | head | body | jaw | x | GU016855 | x |
| Apoyeque | *A.* cf. *citrinellus* | AMCK00812 | N | M | | head | body | jaw | x | GU016856 | x |
| Apoyeque | *A.* cf. *citrinellus* | AMCK00813 | N | M | | | | | x | GU016857 | x |
| Apoyeque | *A.* cf. *citrinellus* | AMCK00814 | N | F | | | | | x | GU016858 | x |
| Apoyeque | *A.* cf. *citrinellus* | AMCK00815 | N | M | | head | body | jaw | x | GU016859 | x |
| Apoyeque | *A.* cf. *citrinellus* | AMCK00816 | N | J | | | | | x | GU016860 | x |
| Apoyeque | *A.* cf. *citrinellus* | AMCK00817 | N | J | | | | | x | GU016861 | x |
| Apoyeque | *A.* cf. *citrinellus* | AMCK00818 | N | not noted | | | | | x | GU016862 | x |

| | | | | | | | | | | | | |
|---|---|---|---|---|---|---|---|---|---|---|---|---|
| Apoyeque | *A.* cf. *citrinellus* | AMCK00819 | N | F | | | head | body | jaw | x | | x |
| Apoyeque | *A.* cf. *citrinellus* | AMCK00820 | N | J | | | | | | x | GU016863 | x |
| Apoyeque | *A.* cf. *citrinellus* | AMCK00821 | L | not noted | | | | | | x | | x |
| Apoyeque | *A.* cf. *citrinellus* | AMCK00822 | N | J | | | | | | x | GU016864 | x |
| Apoyeque | *A.* cf. *citrinellus* | AMCK00823 | N | M | | | | | | x | GU016865 | x |
| Apoyeque | *A.* cf. *citrinellus* | AMCK00824 | N | M | | | head | body | jaw | x | GU016866 | x |
| Apoyeque | *A.* cf. *citrinellus* | AMCK00825 | N | F | | | head | body | jaw | x | GU016867 | x |
| Apoyeque | *A.* cf. *citrinellus* | AMCK00826 | N | M | | | head | body | jaw | x | GU016868 | x |
| Apoyeque | *A.* cf. *citrinellus* | AMCK00827 | L | F | | | head | body | jaw | x | | x |
| Apoyeque | *A.* cf. *citrinellus* | AMCK00828 | N | M | | | | | | x | | x |
| Apoyeque | *A.* cf. *citrinellus* | AMCK00931 | L | F | SIA | gut | | | | | GU016869 | x |
| Apoyeque | *A.* cf. *citrinellus* | AMCK00932 | N | not noted | | | | | | | GU016870 | x |
| Apoyeque | *A.* cf. *citrinellus* | AMCK00933 | L | not noted | SIA | | | | | | GU016871 | x |
| Apoyeque | *A.* cf. *citrinellus* | AMCK00934 | N | F | | | | | | | GU016872 | x |
| Apoyeque | *A.* cf. *citrinellus* | AMCK00935 | L | J | | | | | | | GU016873 | x |
| Apoyeque | *A.* cf. *citrinellus* | AMCK00936 | N | F | | | | | | | GU016874 | x |
| Apoyeque | *A.* cf. *citrinellus* | AMCK00937 | L | F | SIA | gut | | | | | GU016875 | x |
| Apoyeque | *A.* cf. *citrinellus* | AMCK00938 | N | M | | | | | | | GU016876 | x |
| Apoyeque | *A.* cf. *citrinellus* | AMCK00939 | L | F | SIA | gut | | | | | GU016877 | x |
| Apoyeque | *A.* cf. *citrinellus* | AMCK00940 | N | F | SIA | gut | head | body | jaw | x | GU016878 | x |
| Apoyeque | *A.* cf. *citrinellus* | AMCK00941 | N | J | | | | | | x | GU016879 | x |
| Apoyeque | *A.* cf. *citrinellus* | AMCK00942 | N | M | SIA | | head | body | jaw | x | GU016880 | x |
| Apoyeque | *A.* cf. *citrinellus* | AMCK00943 | N | M | SIA | gut | head | body | jaw | x | GU016881 | x |
| Apoyeque | *A.* cf. *citrinellus* | AMCK00944 | N | M | | gut | head | body | jaw | x | GU016882 | x |
| Apoyeque | *A.* cf. *citrinellus* | AMCK00945 | L | M | | gut | | | | x | GU016883 | x |
| Apoyeque | *A.* cf. *citrinellus* | AMCK00946 | N | J | | | | | | x | GU016884 | x |
| Apoyeque | *A.* cf. *citrinellus* | AMCK00947 | L | F | SIA | gut | head | body | jaw | x | GU016885 | x |
| Apoyeque | *A.* cf. *citrinellus* | AMCK00948 | L | F | | gut | head | body | jaw | x | GU016886 | x |
| Apoyeque | *A.* cf. *citrinellus* | AMCK00949 | N | M | | gut | head | body | jaw | x | GU016887 | x |
| Apoyeque | *A.* cf. *citrinellus* | AMCK00950 | L | F | | gut | head | body | jaw | x | GU016888 | x |

| | | | | | | | | | | | | |
|---|---|---|---|---|---|---|---|---|---|---|---|---|
| Apoyeque | *A.* cf. *citrinellus* | AMCK00951 | L | M | | | head | body | jaw | x | GU016889 | x |
| Apoyeque | *A.* cf. *citrinellus* | AMCK00952 | L | not noted | | gut | | | | x | GU016890 | x |
| Apoyeque | *A.* cf. *citrinellus* | AMCK00953 | N | F | | | head | body | jaw | x | GU016891 | x |
| Apoyeque | *A.* cf. *citrinellus* | AMCK00954 | L | F | SIA | | head | body | jaw | x | GU016892 | x |
| Apoyeque | *A.* cf. *citrinellus* | AMCK00955 | L | not noted | SIA | gut | | | | x | GU016893 | x |
| Apoyeque | *A.* cf. *citrinellus* | AMCK00956 | N | M | SIA | gut | head | body | jaw | x | GU016894 | x |
| Apoyeque | *A.* cf. *citrinellus* | AMCK00957 | N | F | SIA | | head | body | jaw | x | GU016895 | x |
| Apoyeque | *A.* cf. *citrinellus* | AMCK00958 | N | M | SIA | gut | head | body | jaw | x | GU016896 | x |
| Apoyeque | *A.* cf. *citrinellus* | AMCK00959 | N | F | SIA | | head | body | jaw | x | GU016897 | x |
| Apoyeque | *A.* cf. *citrinellus* | AMCK00960 | N | F | | | head | body | jaw | x | GU016898 | x |
| Apoyeque | *A.* cf. *citrinellus* | AMCK00961 | N | M | | | head | body | jaw | x | GU016899 | x |
| Apoyeque | *A.* cf. *citrinellus* | AMCK00962 | N | M | SIA | gut | head | body | jaw | x | GU016900 | x |
| Apoyeque | *A.* cf. *citrinellus* | AMCK00963 | N | M | SIA | gut | head | body | jaw | x | GU016901 | x |
| Apoyeque | *A.* cf. *citrinellus* | AMCK00964 | N | J | | | | | | x | GU016902 | x |
| Apoyeque | *A.* cf. *citrinellus* | AMCK00965 | L | F | SIA | gut | head | body | jaw | x | GU016903 | x |
| Apoyeque | *A.* cf. *citrinellus* | AMCK00966 | L | J | | | | | | x | GU016904 | x |
| Apoyeque | *A.* cf. *citrinellus* | AMCK00967 | N | not noted | | | | | | x | GU016905 | x |
| Apoyeque | *A.* cf. *citrinellus* | AMCK00968 | N | F | | | head | body | jaw | x | GU016906 | x |
| Apoyeque | *A.* cf. *citrinellus* | AMCK00969 | N | M | | | head | body | jaw | x | GU016907 | x |
| Apoyeque | *A.* cf. *citrinellus* | AMCK00970 | N | M | | | head | body | jaw | x | GU016908 | x |
| Apoyeque | *A.* cf. *citrinellus* | AMCK00971 | N | J | | | | | | x | | x |
| Apoyeque | *A.* cf. *citrinellus* | AMCK00972 | N | M | | | head | body | jaw | x | GU016909 | x |
| Apoyeque | *A.* cf. *citrinellus* | AMCK00973 | L | not noted | SIA | gut | | | | x | GU016910 | x |
| Apoyeque | *A.* cf. *citrinellus* | AMCK00974 | N | not noted | | gut | | | | x | GU016911 | x |
| Apoyeque | *A.* cf. *citrinellus* | AMCK00975 | N | J | | | | | | x | GU016912 | x |
| Apoyeque | *A.* cf. *citrinellus* | AMCK00976 | N | M | | | head | body | jaw | x | GU016913 | x |
| Apoyeque | *A.* cf. *citrinellus* | AMCK00977 | N | J | | | | | | x | GU016914 | x |
| Apoyeque | *A.* cf. *citrinellus* | AMCK00978 | L | J | | | | | | x | GU016915 | x |
| Apoyeque | *A.* cf. *citrinellus* | AMCK00979 | N | F | | | head | body | jaw | x | | x |
| Apoyeque | *A.* cf. *citrinellus* | AMCK00980 | L | F | SIA | | head | body | jaw | x | GU016916 | x |

| | | | | | | | | | | | | |
|---|---|---|---|---|---|---|---|---|---|---|---|---|
| Apoyeque | *A.* cf. *citrinellus* | AMCK00981 | N | F | SIA | | head | body | jaw | x | GU016917 | x |
| Apoyeque | *A.* cf. *citrinellus* | AMCK00982 | N | M | | | head | body | jaw | x | GU016918 | x |
| Apoyeque | *A.* cf. *citrinellus* | AMCK00983 | N | not noted | | | | | | x | GU016919 | x |
| Apoyeque | *A.* cf. *citrinellus* | AMCK00984 | N | M | | | head | body | jaw | x | GU016920 | x |
| Apoyeque | *A.* cf. *citrinellus* | AMCK00985 | N | M | | | head | body | jaw | x | GU016921 | x |
| Apoyeque | *A.* cf. *citrinellus* | AMCK00986 | N | M | | gut | head | body | jaw | x | GU016922 | x |
| Apoyeque | *A.* cf. *citrinellus* | AMCK00987 | N | J | | | | | | x | GU016923 | x |
| Apoyeque | *A.* cf. *citrinellus* | AMCK00988 | N | M | | gut | head | body | jaw | x | GU016924 | x |
| Apoyeque | *A.* cf. *citrinellus* | AMCK00989 | N | J | | | | | | x | GU016925 | x |
| Apoyeque | *A.* cf. *citrinellus* | AMCK00990 | N | M | | | head | body | jaw | x | GU016926 | x |
| Apoyeque | *A.* cf. *citrinellus* | AMCK00991 | N | J | | | | | | x | GU016927 | x |
| Apoyeque | *A.* cf. *citrinellus* | AMCK00992 | L | not noted | | gut | | | | x | GU016928 | x |
| Apoyeque | *A.* cf. *citrinellus* | AMCK00993 | N | F | | | head | body | jaw | x | GU016929 | x |
| Apoyeque | *A.* cf. *citrinellus* | AMCK00994 | N | M | | | head | body | jaw | x | GU016930 | x |
| Apoyeque | *A.* cf. *citrinellus* | AMCK00995 | N | J | | | | | | x | GU016931 | x |
| Apoyeque | *A.* cf. *citrinellus* | AMCK00996 | N | M | | | | | | x | GU016932 | x |
| Apoyeque | *A.* cf. *citrinellus* | AMCK00997 | N | M | | | head | body | jaw | x | GU016933 | x |
| Apoyeque | *A.* cf. *citrinellus* | AMCK00998 | N | J | | | | | | x | GU016934 | x |
| Apoyeque | *A.* cf. *citrinellus* | AMCK00999 | L | J | | | | | | x | GU016935 | x |
| Apoyeque | *A.* cf. *citrinellus* | AMCK01000 | N | M | SIA | | head | body | jaw | x | GU016936 | x |
| Apoyeque | *A.* cf. *citrinellus* | AMCK01001 | N | J | | | | | | x | GU016937 | x |
| Apoyeque | *A.* cf. *citrinellus* | AMCK01002 | N | F | | | | | | x | GU016938 | x |
| Apoyeque | *A.* cf. *citrinellus* | AMCK01003 | N | J | | | | | | x | GU016939 | x |
| Apoyeque | *A.* cf. *citrinellus* | AMCK01004 | N | J | | | | | | x | GU016940 | x |
| Apoyeque | *A.* cf. *citrinellus* | AMCK01005 | L | F | SIA | gut | head | body | jaw | x | GU016941 | x |
| Apoyeque | *A.* cf. *citrinellus* | AMCK01006 | N | J | | | | | | x | GU016942 | x |
| Apoyeque | *A.* cf. *citrinellus* | AMCK01007 | N | J | | | | | | x | GU016943 | x |
| Apoyeque | *A.* cf. *citrinellus* | AMCK01008 | N | F | | | head | body | jaw | x | GU016944 | x |
| Apoyeque | *A.* cf. *citrinellus* | AMCK01009 | N | J | | | | | | x | GU016945 | x |
| Apoyeque | *A.* cf. *citrinellus* | AMCK01010 | N | F | | | head | body | jaw | x | GU016946 | x |

| | | | | | | | | | | | | |
|---|---|---|---|---|---|---|---|---|---|---|---|---|
| Apoyeque | *A.* cf. *citrinellus* | AMCK01011 | N | F | | | | | | x | GU016947 | x |
| Apoyeque | *A.* cf. *citrinellus* | AMCK01012 | N | not noted | | | | | | x | GU016948 | x |
| Apoyeque | *A.* cf. *citrinellus* | AMCK01013 | N | J | | | | | | x | GU016949 | x |
| Apoyeque | *A.* cf. *citrinellus* | AMCK01014 | L | F | | | head | body | jaw | x | GU016950 | x |
| Apoyeque | *A.* cf. *citrinellus* | AMCK01015 | L | not noted | | | | | | x | GU016951 | x |
| Apoyeque | *A.* cf. *citrinellus* | AMCK01016 | N | M | | | head | body | jaw | x | GU016952 | x |
| Apoyeque | *A.* cf. *citrinellus* | AMCK01017 | N | M | | | head | body | jaw | x | GU016953 | x |
| Apoyeque | *A.* cf. *citrinellus* | AMCK01018 | N | F | | | head | body | jaw | x | GU016954 | x |
| Apoyeque | *A.* cf. *citrinellus* | AMCK01019 | N | F | | | head | body | jaw | x | GU016955 | x |
| Apoyeque | *A.* cf. *citrinellus* | AMCK01020 | L | F | SIA | gut | head | body | jaw | x | GU016956 | x |
| Apoyeque | *A.* cf. *citrinellus* | AMCK01021 | N | J | | | | | | x | GU016957 | x |
| Apoyeque | *A.* cf. *citrinellus* | AMCK01022 | N | J | | | | | | x | GU016958 | x |
| Apoyeque | *A.* cf. *citrinellus* | AMCK01023 | L | M | | | head | body | jaw | x | GU016959 | x |
| Apoyeque | *A.* cf. *citrinellus* | AMCK01024 | N | J | | | | | | x | GU016960 | x |
| Apoyeque | *A.* cf. *citrinellus* | AMCK01025 | N | F | | | head | body | jaw | x | GU016961 | x |
| Apoyeque | *A.* cf. *citrinellus* | AMCK01026 | N | F | | | head | body | jaw | x | GU016962 | x |
| Apoyeque | *A.* cf. *citrinellus* | AMCK01027 | N | M | | | head | body | jaw | x | GU016963 | x |
| Apoyeque | *A.* cf. *citrinellus* | AMCK01028 | N | M | | | | | | x | GU016964 | x |
| Apoyeque | *A.* cf. *citrinellus* | AMCK01029 | N | F | | | head | body | jaw | | GU016965 | x |
| Apoyeque | *A.* cf. *citrinellus* | AMCK01030 | N | M | SIA | | head | body | jaw | x | GU016966 | x |
| Apoyeque | *A.* cf. *citrinellus* | AMCK01031 | N | F | | | | | | x | GU016967 | x |
| Apoyeque | *A.* cf. *citrinellus* | AMCK01032 | N | F | | | head | body | jaw | x | GU016968 | x |
| Apoyeque | *A.* cf. *citrinellus* | AMCK01033 | L | F | | | head | body | jaw | x | GU016969 | x |
| Apoyeque | *A.* cf. *citrinellus* | AMCK01034 | N | F | | | | | | x | GU016970 | x |
| Apoyeque | *A.* cf. *citrinellus* | AMCK01035 | N | M | | | head | body | jaw | x | GU016971 | x |
| Apoyeque | *A.* cf. *citrinellus* | AMCK01036 | N | F | | | head | body | jaw | x | GU016972 | x |
| Apoyeque | *A.* cf. *citrinellus* | AMCK01037 | N | F | | | head | body | jaw | x | GU016973 | x |
| Apoyeque | *A.* cf. *citrinellus* | AMCK01038 | N | F | | | head | body | jaw | x | GU016974 | x |
| Apoyeque | *A.* cf. *citrinellus* | AMCK01039 | L | F | | | head | body | jaw | x | GU016975 | x |
| Apoyeque | *A.* cf. *citrinellus* | AMCK01040 | N | M | | | head | body | jaw | x | GU016976 | x |

| | | | | | | | | | | |
|---|---|---|---|---|---|---|---|---|---|---|
| Apoyeque | *A.* cf. *citrinellus* | AMCK01041 | N | F | head | body | jaw | x | GU016977 | x |
| Apoyeque | *A.* cf. *citrinellus* | AMCK01042 | N | M | head | body | jaw | x | GU016978 | x |
| Apoyeque | *A.* cf. *citrinellus* | AMCK01043 | N | M | head | body | jaw | x | GU016979 | x |
| Apoyeque | *A.* cf. *citrinellus* | AMCK01044 | N | F | head | body | jaw | x | GU016980 | x |
| Apoyeque | *A.* cf. *citrinellus* | AMCK01045 | N | J | | | | x | GU016981 | x |
| Apoyeque | *A.* cf. *citrinellus* | AMCK01046 | L | J | | | | x | GU016982 | x |
| Apoyeque | *A.* cf. *citrinellus* | AMCK01047 | N | M | head | body | jaw | x | GU016983 | x |
| Apoyeque | *A.* cf. *citrinellus* | AMCK01048 | N | F | head | body | jaw | x | GU016984 | x |
| Apoyeque | *A.* cf. *citrinellus* | AMCK01049 | L | F | head | body | jaw | x | GU016985 | x |
| Apoyeque | *A.* cf. *citrinellus* | AMCK01050 | N | M | | | | x | GU016986 | x |
| Apoyeque | *A.* cf. *citrinellus* | AMCK01051 | N | not noted | | | | x | GU016987 | x |
| Apoyeque | *A.* cf. *citrinellus* | AMCK01052 | L | F | head | body | jaw | x | GU016988 | x |
| Apoyeque | *A.* cf. *citrinellus* | AMCK01053 | N | not noted | | | | x | GU016989 | x |
| Apoyeque | *A.* cf. *citrinellus* | AMCK01054 | N | M | head | body | jaw | x | GU016990 | x |
| Apoyeque | *A.* cf. *citrinellus* | AMCK01055 | N | F | | | | x | GU016991 | x |
| Apoyeque | *A.* cf. *citrinellus* | AMCK01056 | N | J | | | | x | | |
| Apoyeque | *A.* cf. *citrinellus* | AMCK01057 | N | not noted | | | | x | GU016992 | x |
| Apoyeque | *A.* cf. *citrinellus* | AMCK01058 | N | not noted | | | | x | GU016993 | x |
| Xiloa | *A. amarillo* | AMCK00001 | | not noted | | | | | | x |
| Xiloa | *A. amarillo* | AMCK00018 | | not noted | | | | | | x |
| Xiloa | *A. amarillo* | AMCK00019 | | not noted | | | | | | x |
| Xiloa | *A. amarillo* | AMCK00020 | | not noted | | | | | | x |
| Xiloa | *A. amarillo* | AMCK00021 | | not noted | | | | | | x |
| Xiloa | *A. amarillo* | AMCK00022 | | not noted | | | | | | x |
| Xiloa | *A. amarillo* | AMCK00023 | | not noted | | | | | | x |
| Xiloa | *A. amarillo* | AMCK00024 | | not noted | | | | | | x |
| Xiloa | *A. amarillo* | AMCK00025 | | not noted | | | | | | x |
| Xiloa | *A. amarillo* | AMCK00026 | | not noted | | | | | | x |
| Xiloa | *A. amarillo* | AMCK00027 | | not noted | | | | | | x |
| Xiloa | *A. amarillo* | AMCK00028 | | not noted | | | | | | x |

| | | | | | |
|---|---|---|---|---|---|
| Xiloa | *A. amarillo* | AMCK00029 | not noted | | x |
| Xiloa | *A.* cf. *citrinellus* | AMCK00030 | J | | x |
| Xiloa | *A. amarillo* | AMCK00534 | not noted | GU017062 | x |
| Xiloa | *A. amarillo* | AMCK00863 | not noted | GU017063 | x |
| Xiloa | *A. amarillo* | AMCK00864 | not noted | GU017064 | x |
| Xiloa | *A. amarillo* | AMCK00865 | not noted | GU017065 | x |
| Xiloa | *A.* cf. *citrinellus* | AMCK00867 | J | GU017090 | x |
| Xiloa | *A. amarillo* | AMCK00868 | not noted | GU017066 | x |
| Xiloa | *A. sagittae* | AMCK00878 | not noted | | x |
| Xiloa | *A. amarillo* | AMCK00886 | not noted | GU017067 | x |
| Xiloa | *A. amarillo* | AMCK00890 | not noted | GU017068 | x |
| Xiloa | *A. xiloaensis* | AMCK00891 | not noted | GU017080 | x |
| Xiloa | *A. xiloaensis* | AMCK00892 | not noted | GU017081 | x |
| Xiloa | *A. xiloaensis* | AMCK00893 | not noted | GU017082 | x |
| Xiloa | *A. xiloaensis* | AMCK00894 | not noted | GU017083 | x |
| Xiloa | *A. xiloaensis* | AMCK00895 | not noted | | x |
| Xiloa | *A. xiloaensis* | AMCK00896 | not noted | GU017084 | x |
| Xiloa | *A. xiloaensis* | AMCK00897 | not noted | GU017085 | x |
| Xiloa | *A. xiloaensis* | AMCK00898 | not noted | GU017086 | x |
| Xiloa | *A. amarillo* | AMCK00900 | not noted | GU017069 | x |
| Xiloa | *A. xiloaensis* | AMCK00902 | not noted | GU017079 | x |
| Xiloa | *A. amarillo* | AMCK00905 | not noted | GU017070 | x |
| Xiloa | *A. amarillo* | AMCK00908 | not noted | | x |
| Xiloa | *A. amarillo* | AMCK00911 | not noted | GU017071 | x |
| Xiloa | *A. xiloaensis* | AMCK00916 | not noted | GU017087 | x |
| Xiloa | *A.* cf. *citrinellus* | AMCK00924 | J | GU017088 | x |
| Xiloa | *A.* cf. *citrinellus* | AMCK00925 | J | GU017089 | x |
| Xiloa | *A. amarillo* | AMCK00930 | not noted | GU017072 | x |
| Xiloa | *A. amarillo* | AMCK01244 | not noted | | x |
| Xiloa | *A. amarillo* | AMCK01247 | not noted | | x |

| | | | | | |
|---|---|---|---|---|---|
| Xiloa | *A. amarillo* | AMCK01251 | not noted | GU017073 | x |
| Xiloa | *A. amarillo* | AMCK01253 | not noted | GU017074 | x |
| Xiloa | *A. amarillo* | AMCK01254 | not noted | | x |
| Xiloa | *A. amarillo* | AMCK01255 | not noted | GU017075 | x |
| Xiloa | *A. amarillo* | AMCK01256 | not noted | GU017076 | x |
| Xiloa | *A. amarillo* | AMCK01257 | not noted | GU017077 | x |
| Xiloa | *A. amarillo* | AMCK01258 | not noted | | x |
| Xiloa | *A. amarillo* | AMCK01259 | not noted | GU017078 | x |
| Managua | *A. citrinellus* | 03022 | not noted | | x |
| Managua | *A. citrinellus* | 03023 | not noted | | x |
| Managua | *A. citrinellus* | 03024 | not noted | | x |
| Managua | *A. citrinellus* | 03025 | not noted | | x |
| Managua | *A. citrinellus* | 03026 | not noted | | x |
| Managua | *A. citrinellus* | 03027 | not noted | | x |
| Managua | *A. citrinellus* | 03028 | not noted | | x |
| Managua | *A. citrinellus* | 03029 | not noted | | x |
| Managua | *A. citrinellus* | 03030 | not noted | | x |
| Managua | *A. citrinellus* | 03031 | not noted | | x |
| Managua | *A. citrinellus* | 03032 | not noted | | x |
| Managua | *A. citrinellus* | 03033 | not noted | | x |
| Managua | *A. citrinellus* | 03034 | not noted | | x |
| Managua | *A. citrinellus* | 03035 | not noted | | x |
| Managua | *A. citrinellus* | 03036 | not noted | | x |
| Managua | *A. citrinellus* | 03037 | not noted | | x |
| Managua | *A. citrinellus* | 03038 | not noted | | x |
| Managua | *A. citrinellus* | 03039 | not noted | | x |
| Managua | *A. citrinellus* | 03040 | not noted | | x |
| Managua | *A. citrinellus* | 03041 | not noted | | x |
| Managua | *A. citrinellus* | 03042 | not noted | | x |
| Managua | *A. citrinellus* | 03043 | not noted | | x |

| | | | | |
|---|---|---|---|---|
| Managua | *A. citrinellus* | 03044 | not noted | x |
| Managua | *A. citrinellus* | 03045 | not noted | x |
| Managua | *A. citrinellus* | 03046 | not noted | x |
| Managua | *A. citrinellus* | 03047 | not noted | x |
| Managua | *A. citrinellus* | 03048 | not noted | x |
| Managua | *A. citrinellus* | 03049 | not noted | x |
| Managua | *A. citrinellus* | 03050 | not noted | x |
| Managua | *A. citrinellus* | 03051 | not noted | x |
| Managua | *A. citrinellus* | 03052 | not noted | x |
| Managua | *A. citrinellus* | 03053 | not noted | x |
| Managua | *A. citrinellus* | 03054 | not noted | x |
| Managua | *A. citrinellus* | 03055 | not noted | x |
| Managua | *A. citrinellus* | 03056 | not noted | x |
| Managua | *A. citrinellus* | 03057 | not noted | x |
| Managua | *A. citrinellus* | 03058 | not noted | x |
| Managua | *A. citrinellus* | 03059 | not noted | x |
| Managua | *A. citrinellus* | 03060 | not noted | x |
| Managua | *A. citrinellus* | 03061 | not noted | x |
| Managua | *A. citrinellus* | 03062 | not noted | x |
| Managua | *A. citrinellus* | 03063 | not noted | x |
| Managua | *A. citrinellus* | 03064 | not noted | x |
| Managua | *A. citrinellus* | 03066 | not noted | x |
| Managua | *A. citrinellus* | 03067 | not noted | x |
| Managua | *A. citrinellus* | 03068 | not noted | x |
| Managua | *A. citrinellus* | 03069 | not noted | x |
| Managua | *A. citrinellus* | 03070 | not noted | x |
| Managua | *A. citrinellus* | 03071 | not noted | x |
| Managua | *A. citrinellus* | 03072 | not noted | x |
| Managua | *A. citrinellus* | 03073 | not noted | x |
| Managua | *A. citrinellus* | 03074 | not noted | x |

| | | | | |
|---|---|---|---|---|
| Managua | *A. citrinellus* | 03075 | not noted | x |
| Managua | *A. citrinellus* | 03924 | not noted | x |
| Managua | *A. citrinellus* | 03925 | not noted | x |
| Managua | *A. citrinellus* | 03926 | not noted | x |
| Managua | *A. citrinellus* | 03927 | not noted | x |
| Managua | *A. citrinellus* | 03928 | not noted | x |
| Managua | *A. citrinellus* | 03929 | not noted | x |
| Managua | *A. citrinellus* | 05001 | not noted | x |
| Managua | *A. labiatus* | 05002 | not noted | x |
| Managua | *A. labiatus* | 05249 | not noted | x |
| Managua | *A. labiatus* | 05250 | not noted | x |
| Managua | *A. labiatus* | 05251 | not noted | x |
| Managua | *A. citrinellus* | 05333 | not noted | x |
| Managua | *A. citrinellus* | 05334 | not noted | x |
| Managua | *A. citrinellus* | 05335 | not noted | x |
| Managua | *A. labiatus* | 05598 | not noted | x |
| Managua | *A. labiatus* | 05599 | not noted | x |
| Managua | *A. labiatus* | 05600 | not noted | x |
| Managua | *A. labiatus* | 05601 | not noted | x |
| Managua | *A. labiatus* | 05602 | not noted | x |
| Managua | *A. labiatus* | 05603 | not noted | x |
| Managua | *A. labiatus* | 05604 | not noted | x |
| Managua | *A. labiatus* | 05605 | not noted | x |
| Managua | *A. labiatus* | 05606 | not noted | x |
| Managua | *A. labiatus* | 05607 | not noted | x |
| Managua | *A. labiatus* | 05814 | not noted | x |
| Managua | *A. labiatus* | 05815 | not noted | x |
| Managua | *A. labiatus* | 05816 | not noted | x |
| Managua | *A. labiatus* | 05817 | not noted | x |
| Managua | *A. labiatus* | 05818 | not noted | x |

| | | | | | |
|---|---|---|---|---|---|
| Managua | *A. labiatus* | 05819 | not noted | | x |
| Managua | *A. labiatus* | 05820 | not noted | | x |
| Managua | *A. labiatus* | 05821 | not noted | | x |
| Managua | *A. labiatus* | 05822 | not noted | | x |
| Managua | *A. labiatus* | 05823 | not noted | | x |
| Managua | *A. labiatus* | 05824 | not noted | | x |
| Managua | *A. labiatus* | 05825 | not noted | | x |
| Managua | *A. labiatus* | 05826 | not noted | | x |
| Managua | *A. labiatus* | 05827 | not noted | | x |
| Managua | *A. labiatus* | 05828 | not noted | | x |
| Managua | *A. citrinellus* | 03 0001 | not noted | HM183496 | x |
| Managua | *A. citrinellus* | 03 0002 | not noted | HM183497 | x |
| Managua | *A. citrinellus* | 03 0003 | not noted | HM183498 | x |
| Managua | *A. citrinellus* | 03 0004 | not noted | | x |
| Managua | *A. citrinellus* | 03 0006 | not noted | HM183501 | x |
| Managua | *A. citrinellus* | 03 0007 | not noted | HM183502 | x |
| Managua | *A. citrinellus* | 03 0008 | not noted | | x |
| Managua | *A. citrinellus* | 03 0009 | not noted | HM183504 | x |
| Managua | *A. citrinellus* | 03 0010 | not noted | | x |
| Managua | *A. citrinellus* | 03 0011 | not noted | HM183506 | x |
| Managua | *A. citrinellus* | 03 0012 | not noted | HM183507 | x |
| Managua | *A. citrinellus* | 03 0013 | not noted | HM183508 | x |
| Managua | *A. citrinellus* | 03 0014 | not noted | HM183509 | x |
| Managua | *A. citrinellus* | 03 0015 | not noted | HM183509 | x |
| Managua | *A. citrinellus* | 03 0016 | not noted | HM183511 | x |
| Managua | *A. citrinellus* | 03 0017 | not noted | HM204814 | x |
| Managua | *A. citrinellus* | 03 0018 | not noted | HM183512 | x |
| Managua | *A. citrinellus* | 03 0019 | not noted | HM183513 | x |
| Managua | *A. citrinellus* | 03 0020 | not noted | HM183514 | x |
| Managua | *A. citrinellus* | 03 0021 | not noted | HM183515 | x |

| | | | | | |
|---|---|---|---|---|---|
| Managua | *A. citrinellus* | 03 0076 | not noted | HM183570 | x |
| Managua | *A. citrinellus* | 03 0077 | not noted | HM183571 | x |
| Managua | *A. citrinellus* | 03 0078 | not noted | HM183572 | x |
| Managua | *A. citrinellus* | 03 0079 | not noted | HM183573 | x |
| Managua | *A. citrinellus* | 03 0080 | not noted | HM183574 | x |
| Managua | *A. citrinellus* | 03 0081 | not noted | HM183575 | x |
| Managua | *A. citrinellus* | 03 0082 | not noted | HM183576 | x |
| Managua | *A. citrinellus* | 03 0083 | not noted | HM183577 | x |
| Managua | *A. citrinellus* | 03 0084 | not noted | HM183578 | x |
| Managua | *A. citrinellus* | 03 0085 | not noted | | x |
| Managua | *A. citrinellus* | 03 0930 | not noted | HM184423 | x |
| Managua | *A. citrinellus* | 03 0931 | not noted | HM184424 | x |
| Managua | *A. citrinellus* | 03 0933 | not noted | HM184426 | x |
| Managua | *A. citrinellus* | 03 0934 | not noted | HM184427 | x |
| Managua | *A. citrinellus* | 03 0935 | not noted | HM204815 | x |
| Managua | *A. citrinellus* | 03 0936 | not noted | HM184429 | x |
| Managua | *A. citrinellus* | 03 0937 | not noted | | x |
| Managua | *A. citrinellus* | 03 0938 | not noted | | x |
| Managua | *A. citrinellus* | 03 0939 | not noted | HM184432 | x |
| Managua | *A. citrinellus* | 03 0940 | not noted | | x |
| Managua | *A. citrinellus* | 03 0941 | not noted | HM184434 | x |
| Managua | *A. citrinellus* | 03 0942 | not noted | HM184435 | x |
| Managua | *A. citrinellus* | 03 0943 | not noted | HM184436 | x |
| Managua | *A. citrinellus* | 03 0944 | not noted | HM184437 | x |
| Managua | *A. citrinellus* | 03 0945 | not noted | HM184438 | x |
| Managua | *A. citrinellus* | 03 0946 | not noted | HM184439 | x |
| Managua | *A. citrinellus* | 03 0947 | not noted | HM184440 | x |
| Managua | *A. citrinellus* | 03 0948 | not noted | | x |
| Managua | *A. citrinellus* | 03 0949 | not noted | HM184442 | x |
| Managua | *A. citrinellus* | 03 0950 | not noted | | x |

| | | | | | |
|---|---|---|---|---|---|
| Managua | *A. citrinellus* | 03 0951 | not noted | HM184444 | x |
| Managua | *A. citrinellus* | 03 0952 | not noted | HM184445 | x |
| Managua | *A. citrinellus* | 03 0953 | not noted | | x |
| Managua | *A. citrinellus* | 03 0954 | not noted | HM184447 | x |
| Managua | *A. citrinellus* | 03 0955 | not noted | HM184448 | x |
| Managua | *A. citrinellus* | 03 0956 | not noted | HM184449 | x |
| Managua | *A. citrinellus* | 03 0957 | not noted | HM184450 | x |
| Managua | *A. citrinellus* | 03 0958 | not noted | HM184451 | x |
| Managua | *A. citrinellus* | 03 0959 | not noted | HM184452 | x |
| Managua | *A. labiatus* | 03 1067 | not noted | | x |
| Managua | *A. labiatus* | 03 1068 | not noted | HM184561 | x |
| Managua | *A. labiatus* | 03 1069 | not noted | HM184562 | x |
| Managua | *A. citrinellus* | 436 | not noted | HM204816 | x |
| Managua | *A. citrinellus* | 447 | not noted | | x |
| Managua | *A. citrinellus* | 448 | not noted | AY567337 | x |
| Managua | *A. citrinellus* | 473 | not noted | | x |
| Managua | *A. citrinellus* | 474 | not noted | | x |
| Managua | *A. citrinellus* | 475 | not noted | | x |
| Managua | *A. citrinellus* | 476 | not noted | | x |
| Managua | *A. citrinellus* | 477 | not noted | HM204817 | x |
| Managua | *A. citrinellus* | 478 | not noted | | x |
| Managua | *A. citrinellus* | 479 | not noted | AY567361 | x |
| Managua | *A. citrinellus* | 480 | not noted | HM204818 | x |
| Managua | *A. citrinellus* | 481 | not noted | AY567362 | x |
| Managua | *A. citrinellus* | 482 | not noted | AY567363 | x |
| Managua | *A. citrinellus* | 484 | not noted | HM204819 | x |
| Managua | *A. citrinellus* | 485 | not noted | HM204820 | x |
| Managua | *A. citrinellus* | 486 | not noted | HM204821 | x |
| Managua | *A. citrinellus* | 487 | not noted | AY567364 | x |
| Managua | *A. citrinellus* | 488 | not noted | | x |

| | | | | | |
|---|---|---|---|---|---|
| Managua | *A. citrinellus* | 489 | not noted | AY567366 | x |
| Managua | *A. citrinellus* | 490 | not noted | AY567367 | x |
| Managua | *A. citrinellus* | 491 | not noted | AY567368 | x |
| Managua | *A. citrinellus* | 492 | not noted | AY567369 | x |
| Managua | *A. citrinellus* | 493 | not noted | AY567370 | x |
| Managua | *A. citrinellus* | 494 | not noted | | x |
| Managua | *A. citrinellus* | 495 | not noted | | x |
| Managua | *A. citrinellus* | 496 | not noted | | x |
| Managua | *A. citrinellus* | 497 | not noted | | x |
| Managua | *A. citrinellus* | 498 | not noted | | x |
| Managua | *A. citrinellus* | 499 | not noted | HM204822 | x |
| Managua | *A. citrinellus* | 500 | not noted | AY567374 | x |
| Managua | *A. citrinellus* | 501 | not noted | AY567375 | x |
