# Supplementary material for: Rapid sympatric ecological differentiation of crater lake cichlid fishes within historic times
Source: BMC Biol. 2010 May 12;8:60. doi: 10.1186/1741-7007-8-60 (PMC2880021; doi:10.1186/1741-7007-8-60)
Supplement: Additional file 6 — Standard body length of Midas cichlids in Apoyeque by lip morphology and sex. Body size, measured as standard length, did not differ between thin- and thick-lipped individuals (Two factor ANOVA, morph effect, F1,202 = 0.652, P = 0.42). Males were significantly larger than females (sex effect, F1,202 = 19.9, P < 0.001) for both morphs (morph × sex interaction, F1,202 = 0.082, P = 0.78). [file 1741-7007-8-60-S6.pdf]

Additional File 6: Standard length of mature Midas cichlids in Apoyeque by lip morphology and sex.

Body size, measured as standard length, did not differ between thin- and thick-lipped individuals (Two factor ANOVA, morph effect, $F_{1,202} = 0.652$, $p = 0.42$). Males were significantly larger than females (sex effect, $F_{1,202} = 19.9$, $p < 0.001$) for both morphs (morph × sex interaction, $F_{1,202} = 0.082$, $p = 0.78$).

Lip morph	Sex	*N*	Mean length (cm) ± standard error
thick-lipped	♀	24	9.3 ± 1.8
thick-lipped	♂	15	10.5 ± 5.0
thin-lipped	♀	82	9.5 ± 1.1
thin-lipped	♂	85	10.8 ± 2.1
