# Supplementary material for: Rapid sympatric ecological differentiation of crater lake cichlid fishes within historic times
Source: BMC Biol. 2010 May 12;8:60. doi: 10.1186/1741-7007-8-60 (PMC2880021; doi:10.1186/1741-7007-8-60)

Additional File 7: Bivariate analysis of lip size by body length (mm), fitted with quadratic line for thin- and thick-lipped fishes.

A plot of lip size (total lip area, mm^2) by body length (standard length, mm) with quadratic lines of best-fit shows the different relationship of lip and body size in each of the morphs (n = 309 thin-lipped, n = 71 thick-lipped). A quadratic line (df = 2) had improved goodness-of-fit to the data over a linear (df = 1) regression (thin-lipped: adjusted R^2 = 0.705 *vs*. 0.672, thick-lipped: adjusted R^2 0.506 *vs*. 0.461; non-transformed data).

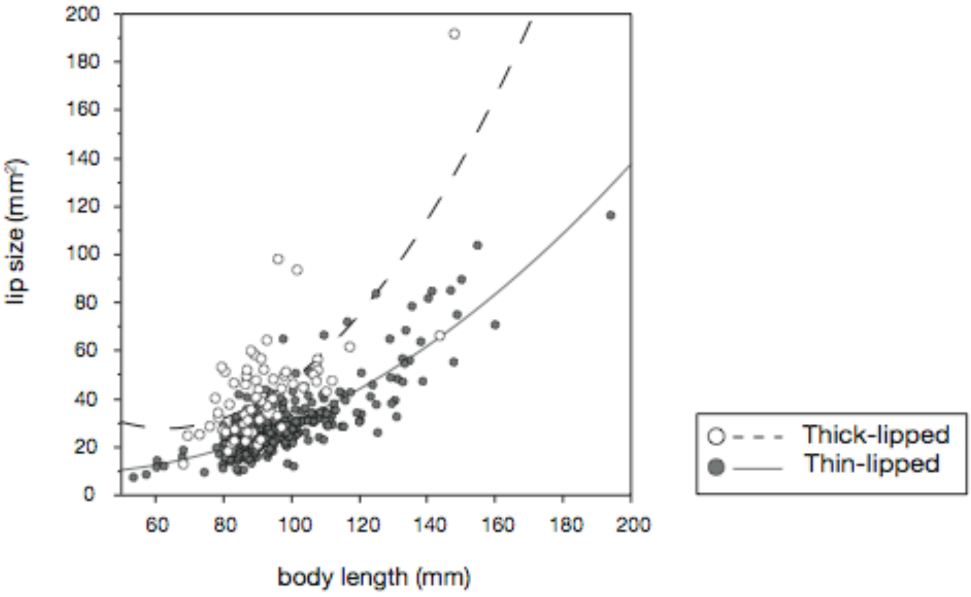

Supplement: Additional file 7 — Bivariate analysis of lip size by body length (mm), fitted with quadratic line for thin- and thick-lipped fishes. A plot of lip size (total lip area, mm2) by body length (standard length, mm) with quadratic lines of best-fit shows the different relationship of lip and body size in each of the morphs (n = 309 thin-lipped, n = 71 thick-lipped). A quadratic (df = 2) had improved goodness-of-fit to the data over a linear (df = 1) regression (thin-lipped: adjusted R2 = 0.705 vs. 0.672, thick-lipped: adjusted R2 0.506 vs. 0.461; non-transformed data). [file 1741-7007-8-60-S7.pdf]
